# *Arabidopsis thaliana* alpha1,2-glucosyltransferase (ALG10) is required for efficient N-glycosylation and leaf growth

**DOI:** 10.1111/j.1365-313X.2011.04688.x

**Published:** 2011-07-27

**Authors:** Akhlaq Farid, Martin Pabst, Jennifer Schoberer, Friedrich Altmann, Josef Glössl, Richard Strasser

**Affiliations:** 1 Department of Applied Genetics and Cell Biology, BOKU-University of Natural Resources and Life SciencesMuthgasse 18, A-1190 Vienna, Austria; 2 Department of Chemistry, BOKU-University of Natural Resources and Life SciencesMuthgasse 18, A-1190 Vienna, Austria

**Keywords:** protein glycosylation, glycosyltransferase, lipid-linked oligosaccharides, posttranslational modification, endoplasmic reticulum, abiotic stress

## Abstract

Assembly of the dolichol-linked oligosaccharide precursor (Glc_3_Man_9_GlcNAc_2_) is highly conserved among eukaryotes. In contrast to yeast and mammals, little is known about the biosynthesis of dolichol-linked oligosaccharides and the transfer to asparagine residues of nascent polypeptides in plants. To understand the biological function of these processes in plants we characterized the *Arabidopsis thaliana* homolog of yeast ALG10, the α1,2-glucosyltransferase that transfers the terminal glucose residue to the lipid-linked precursor. Expression of an Arabidopsis ALG10–GFP fusion protein in *Nicotiana benthamiana* leaf epidermal cells revealed a reticular distribution pattern resembling endoplasmic reticulum (ER) localization. Analysis of lipid-linked oligosaccharides showed that Arabidopsis ALG10 can complement the yeast *Δalg10* mutant strain. A homozygous Arabidopsis T-DNA insertion mutant (*alg10-1*) accumulated mainly lipid-linked Glc_2_Man_9_GlcNAc_2_ and displayed a severe protein underglycosylation defect. Phenotypic analysis of *alg10-1* showed that mutant plants have altered leaf size when grown in soil. Moreover, the inactivation of ALG10 in Arabidopsis resulted in the activation of the unfolded protein response, increased salt sensitivity and suppression of the phenotype of α-glucosidase I-deficient plants. In summary, these data show that Arabidopsis ALG10 is an ER-resident α1,2-glucosyltransferase that is required for lipid-linked oligosaccharide biosynthesis and subsequently for normal leaf development and abiotic stress response.

## Introduction

**Figure 1:**
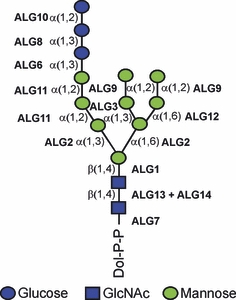
Structure of the lipid-linked Glc_3_Man_9_GlcNAc_2_ oligosaccharide precursor. The glycosyltransferases (ALGs) involved in the biosynthesis of the dolichol-linked (Dol) precursor and the corresponding linkage of the sugar residues are shown.

In eukaryotes, asparagine-linked glycosylation is a common co- and post-translational protein modification. In the first step the pre-assembled core oligosaccharide (Glc_3_Man_9_GlcNAc_2_) is transferred from the dolichyl-pyrophosphate precursor to asparagine residues in Asn-X-Ser/Thr (X cannot be Pro) sequences of nascent polypeptide chains in the lumen of the endoplasmic reticulum (ER). The *N*-glycans are then subjected to a series of highly coordinated step-by-step enzymatic conversions occurring in the ER and Golgi apparatus (Pattison and Amtmann, 2009; Schoberer and Strasser, 2011). The assembly of the dolichyl-pyrophosphate precursor oligosaccharide (Glc_3_Man_9_GlcNAc_2_-PP-Dol) is not well described in plants. However, it has been suggested that the biosynthetic steps and enzymes involved are conserved between humans, yeast and plants (Burda and Aebi, 1999; Lehle *et al.*, 2006). In mammals and yeast, the first biosynthesis step is catalyzed at the cytosolic side of the ER by dolichyl-phosphate GlcNAc-1-phosphotransferase, which adds a single GlcNAc residue to the lipid carrier. This initial step of lipid-linked oligosaccharide assembly can be specifically inhibited by tunicamycin (Koizumi *et al.*, 1999), which subsequently results in severe underglycosylation of proteins and the activation of the unfolded protein response (UPR) (D’Amico *et al.*, 1992; Denecke *et al.*, 1991). After attachment of a second GlcNAc residue, five mannose residues are added from GDP-mannose by mannosyltransferases to build a Man_5_GlcNAc_2_-PP-Dol structure, which is subsequently translocated into the ER. Further elongation occurs in the lumen of the ER by transfer of mannose and glucose residues from dolichol-P-mannose and dolichol-P-glucose to build up the Glc_3_Man_9_GlcNAc_2_-PP-Dol (Burda and Aebi, 1999) (Figure 1). The fully assembled oligosaccharide is then transferred *en bloc* to asparagine residues of nascent polypeptides by the oligosaccharyltransferase complex (Kelleher and Gilmore, 2006).

Processing of the Glc_3_Man_9_GlcNAc_2_ oligosaccharide starts immediately after the transfer by α-glucosidase I (GCSI) that specifically cleaves off the terminal α1,2-linked glucose residue (Helenius and Aebi, 2001; Spiro, 2000). Although the enzymatic properties of *Arabidopsis thaliana* GCSI have not been described so far the *knf-14* mutant, which has a premature stop codon due to the loss of a donor splice site, completely lacks the corresponding α-glucosidase activity (Gillmor *et al.*, 2002). Moreover, GCSI T-DNA insertion mutants (*gcs1*) lacked any processed complex *N*-glycans and instead accumulated Glc_3_Man_7-8_GlcNAc_2_ structures on glycoproteins (Boisson *et al.*, 2001). Importantly, all GCSI-null mutants (*knf-14* and *gcs1*) were described to be embryo lethal, revealing the importance of *N*-glycan processing for cell differentiation and embryo development in plants. Recently a novel *gcsI* (*knf-101*) allele was identified in a screen for genes involved in epidermal development in *A. thaliana* (Furumizu and Komeda, 2008). The *knf-101* mutant, which has a Gly-to-Asp substitution at amino acid residue 504 of GCSI, displays a semi-dwarf phenotype with altered cell shape of the outer epidermal cells in fruits and short and hairy roots (Furumizu and Komeda, 2008). In contrast to *knf-14*/*gcs1* mutants, embryo development was not affected in *knf-101* and the plants were viable and fertile.

The second *N*-glycan processing step is the removal of the two α1,3-linked Glc residues by α-glucosidase II (GCSII). As in mammals, plant GCSII is a heterodimer consisting of the catalytically active α-subunit and the β-subunit, which participates in ER retention of the GCSII α-subunit and assists in deglucosylation (D’Alessio *et al.*, 2010). There is strong evidence that disruption of the Arabidopsis GCSII-α function is lethal, while weaker alleles display a temperature-sensitive phenotype or are more susceptible to pathogen perception (Burn *et al.*, 2002; Lu *et al.*, 2009; Soussilane *et al.*, 2009). On the other hand, null mutants of class I α-mannosidases (MNS1-3), which catalyze the subsequent *N*-glycan trimming reactions are viable and show a developmental phenotype with cell wall alterations under normal growth conditions (Liebminger *et al.*, 2009). Together, these data highlight the importance of the glucose residues for the viability and development of plants. To examine the crucial role of the individual glucose residues in detail we identified the gene homologous to the yeast *ALG10* (asparagine-linked glycosylation) locus, which encodes an α1,2-glucosyltransferase catalyzing the transfer of the terminal glucose residue to generate the fully assembled Glc_3_Man_9_GlcNAc_2_-PP-Dol precursor. Here, we characterized an Arabidopsis *alg10* mutant that displays an underglycosylation defect and altered leaf size under normal growth conditions and reduced tolerance to salt stress. Importantly, the ALG10-deficient plants are viable and suppress the embryo lethality of *knf-14* and the developmental phenotype of the weak *knf-101* mutant. Our results show that efficient glycosylation is required for proper leaf development in plants and suggests that the embryo lethality of *knf-14* is due to an indirect effect caused by a block of further *N*-glycan processing.

## Results

### Identification of the Arabidopsis *ALG10* gene

To identify the putative Arabidopsis α1,2-glucosyltransferase that catalyzes the final glucosylation step during the biosynthesis of the dolichol-linked oligosaccharide precursor (Figure 1) we used the amino acid sequence of the *Saccharomyces cerevisiae* ALG10 (Burda and Aebi, 1998) and performed a BLASTP search in the *A. thaliana* protein database. As a result of this search we identified a single protein encoded by the *At5g02410* gene. This protein has been annotated to the glycosyltransferase family GT59 in the Carbohydrate-Active-enZYmes database (CAZY; http://www.cazy.org/), which contains inverting enzymes that transfer glucose residues from dolichol-P-glucose in α1,2-linkage to Glc_2_Man_9_GlcNAc_2_-PP-Dol, the ultimate step in the assembly of the oligosaccharide precursor. We amplified the whole open reading frame including additional 5′- and 3′-untranslated regions of the Arabidopsis *ALG10* from leaf cDNA. The sequence of the open reading frame was identical to the annotated one from the TAIR database and encodes a protein of 509 amino acid residues. The Arabidopsis ALG10 has 26% identity (44% similarity) to the *S. cerevisiae* ALG10 amino acid sequence (Figure S1 in Supporting Information). It contains three putative N-glycosylation sites and bioinformatic analysis (Plant Protein Membrane Database, http://aramemnon.botanik.uni-koeln.de/) predicts the presence of 12 transmembrane helices (Figure S1) with both ends facing the cytosol as has been suggested for yeast ALG10 (Oriol *et al.*, 2002). Consistent with yeast ALG10, the Arabidopsis homolog does not contain any C-terminal dilysine motif, which can typically be found in other ER-located yeast and Arabidopsis ALG proteins (Oriol *et al.*, 2002; Henquet *et al.*, 2008; Hong *et al.*, 2009; Kajiura *et al.*, 2010), and acts as a Golgi-to-ER-retrieval signal for these proteins. The gene expression profiles from the Bio-Array Resource for Plant Functional Genomics (BAR; http://bbc.botany.utoronto.ca/efp/cgi-bin/efpWeb.cgi) and Genevestigator (https://www.genevestigator.com/gv/index.jsp) indicate that ALG10 expression is high in roots, stems and leaves and reduced in pollen, embryos and endosperm.

**Figure 2:**
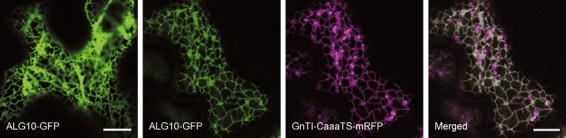
ALG10–GFP displays endoplasmic reticulum (ER) distribution. *Nicotiana benthamiana* leaf epidermal cells expressing the ALG10–GFP fusion protein either alone (left panel) or in combination with the ER-retained construct GnTI-CaaaTS-mRFP. The reticulate fluorescence pattern of ALG10–GFP and its co-localization with GnTI-CaaaTS-mRFP indicate accumulation in the ER. Analysis of fluorescent proteins was done by confocal laser scanning microscopy. Scale bars = 10 μm.

To determine its subcellular localization ALG10 was fused to GFP and transiently expressed in *N. benthamiana* leaf epidermal cells. Analysis of the ALG10–GFP fusion protein by confocal laser scanning microscopy revealed a reticular distribution pattern resembling ER localization (Figure 2). To confirm the localization, we co-expressed ALG10–GFP with the ER-retained GnTI-CaaaTS-mRFP, a mutated fusion protein that mainly localizes to the ER with a minor portion concentrating in the Golgi (Figure 2) (Schoberer *et al.*, 2009). Most of ALG10–GFP displayed co-localization with GnTI-CaaaTS-mRFP, which is in agreement with the proposed function of the enzyme in the assembly of the dolichol-linked oligosaccharide precursor in the ER.

### Arabidopsis ALG10 can complement the yeast *Δalg10* mutant

**Figure 3:**
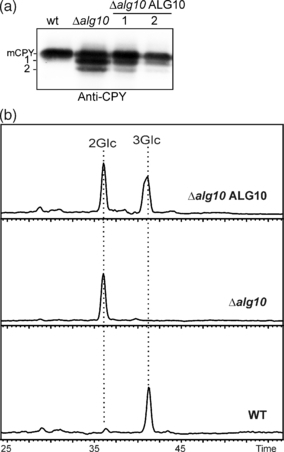
Arabidopsis ALG10 can complement the yeast *Δalg10* mutant. (a) Immunoblot analysis of the CPY glycosylation status. Protein extracts from the *Saccharomyces cerevisiae* wild-type strain BY4741 (wt), the ALG10-deficient yeast strain YGR227W (*Δalg10*) and YGR227W transformed with the plasmid expressing Arabidopsis ALG10 (*Δalg10* ALG10, 1 and 2 represent two independent transformation events) were separated by SDS-PAGE and analyzed by immunoblotting with anti-CPY antibody. The position of mature CPY (mCPY) and the different CPY forms lacking one (1) or two (2) glycans are indicated. (b) Lipid-linked oligosaccharide analysis of the different yeast strains. Selected ion current chromatograms are shown. Glc2 indicates the elution position of Glc_2_Man_9_GlcNAc_2_ and Glc3 the position of Glc_3_Man_9_GlcNAc_2_ structures. All samples contained less intense peaks of smaller structures, which are not shown here.

To determine whether ALG10 is a functional ortholog of the yeast ALG10 glycosyltransferase we expressed the full-length Arabidopsis *ALG10* open reading frame under the control of a constitutive promoter in the *S. cerevisiae Δalg10* knockout strain and tested for complementation of the mutant phenotype. In yeast, ALG10 deficiency results in severe underglycosylation of N-linked glycoproteins because the oligosaccharyltransferase transfers incompletely assembled oligosaccharides with reduced efficiency (Burda and Aebi, 1998). The hypoglycosylation of proteins can be monitored by immunoblotting using antibodies against the vacuolar protease carboxypeptidase Y (CPY). Yeast CPY carries four N-linked glycans and in the *Δalg10* strain two faster-migrating CPY-forms with a reduced number of N-glycans are detected. As shown in Figure 3(a), expression of Arabidopsis ALG10 in *Δalg10* resulted in a reduced number of faster-migrating CPY-forms indicating partial rescue of the CPY underglycosylation defect.

To obtain further evidence for the functionality of Arabidopsis ALG10 we analyzed the restoration of the lipid-linked oligosaccharide defect of the *S. cerevisiae Δalg10* strain. The lipid-linked oligosaccharides were isolated from microsomal fractions, hydrolyzed and analyzed by liquid chromatography–electrospray ionization–mass spectrometry (LC-ESI-MS) analysis. In contrast to wild-type cells, which accumulated a peak corresponding to the fully-assembled Glc_3_Man_9_GlcNAc_2_ precursor, the *Δalg10* mutant displayed a major peak representing Glc_2_Man_9_GlcNAc_2_ (Figure 3b) and smaller amounts of Glc_1_Man_9_GlcNAc_2_ and Man_9_GlcNAc_2_ (data not shown) (Burda and Aebi, 1998). The *Δalg10* yeast strain expressing Arabidopsis ALG10 accumulated a peak that co-eluted with Glc_3_Man_9_GlcNAc_2_. These data show that Arabidopsis ALG10 can restore the lipid-linked oligosaccharide biosynthesis defect of the *Δalg10* mutant yeast strain, indicating that it is the corresponding plant α1,2-glucosyltransferase.

### The *alg10-1* mutant displays a defect in lipid-linked oligosaccharide synthesis

**Figure 4:**
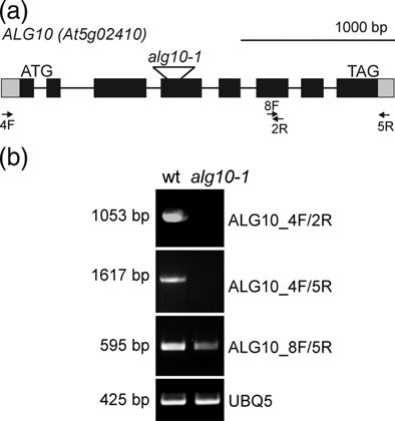
*alg10-1* displays no functional *ALG10* transcript. (a) Schematic overview of the *ALG10* gene structure. Boxes represent exons (the black area represents the coding region), the T-DNA insertion and the primers used (small arrows) are indicated. (b) Reverse transcription-PCR analysis of the *alg10-1* mutant. Reverse transcription-PCR (two independent repeats) was performed on RNA isolated from rosette leaves of Col-0 (wt) and *alg10-1*. Primers specific for the indicated transcripts were then used for amplification. *UBQ5* served as a positive control.

The ER localization and the complementation of the yeast *Δalg10* strain strongly indicate that ALG10 is involved in the assembly of the lipid-linked oligosaccharide precursor. To investigate the *in vivo* function of ALG10 we isolated a homozygous T-DNA insertion line. Sequence analysis showed that the T-DNA insertion in *alg10-1* results in a small deletion of a sequence fragment from exon 4 of the *ALG10* gene (Figure 4a). Reverse transcriptase PCR with different primer combinations confirmed the absence of a functional full-length transcript, indicating that *alg10-1* represents a null allele (Figure 4b).

**Figure 5:**
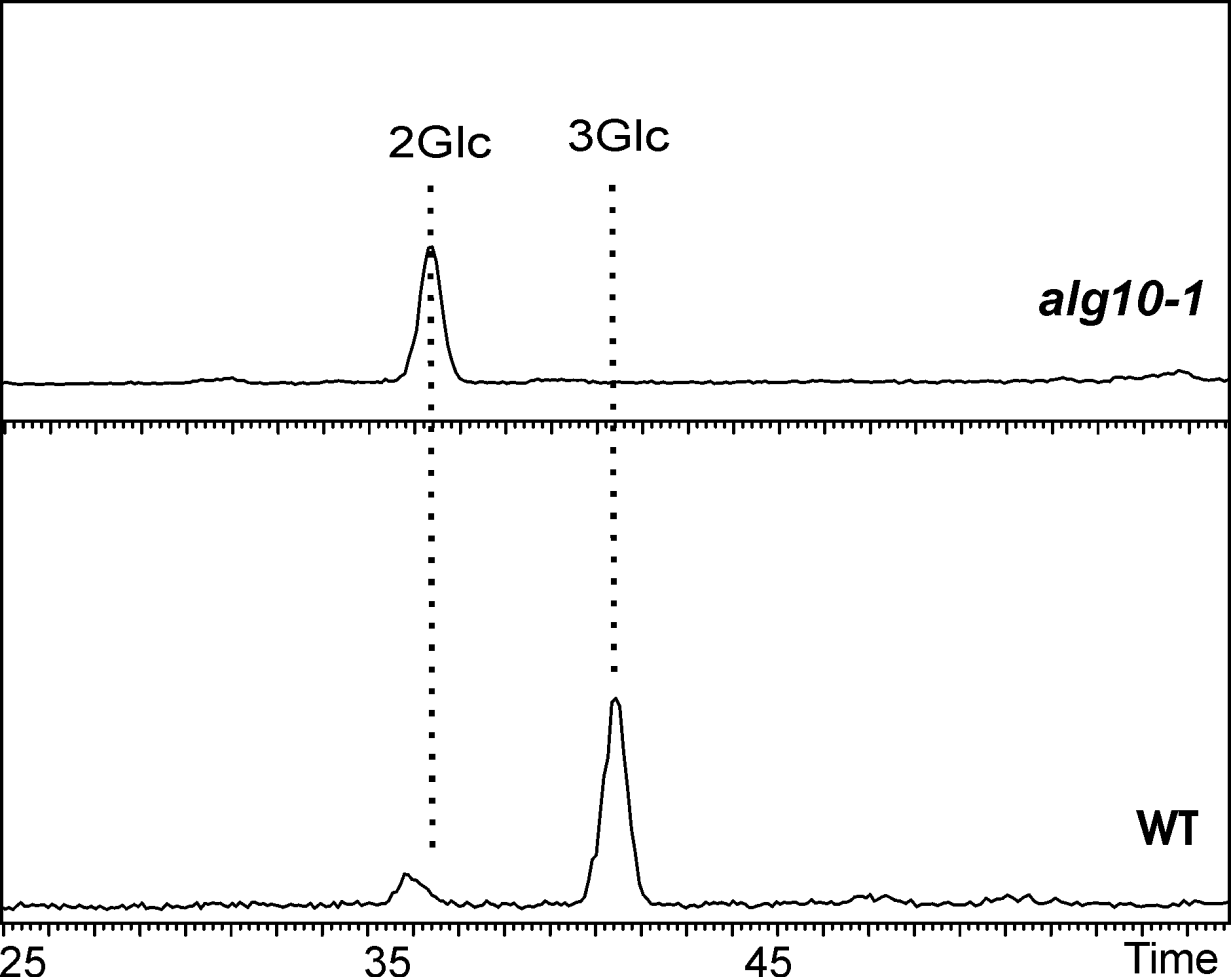
*alg10-1* displays incomplete lipid-linked oligosaccharides. Selected ion current chromatograms for Glc_2_Man_9_GlcNAc_2_ (Glc2) and Glc_3_Man_9_GlcNAc_2_ (Glc3) obtained from lipid-linked oligosaccharides isolated from *Arabidopsis thaliana* seedlings (*alg10-1* and wild-type, WT) and analyzed by liquid chromatography–electrospray ionization–mass spectrometry (LC-ESI-MS). Glc2 indicates the elution position of Glc_2_Man_9_GlcNAc_2_ and Glc3 the position of Glc_3_Man_9_GlcNAc_2_ structures. Both samples contained less intense peaks of smaller structures, which are not shown here.

The effect of ALG10 deficiency on the synthesis of the lipid-linked oligosaccharide precursor in plants was determined by LC-ESI-MS analysis. In wild-type plants the main peak was derived from fully assembled lipid-linked Glc_3_Man_9_GlcNAc_2_. In accordance with the proposed function of ALG10, the *alg10-1* mutant completely lacked this peak and instead accumulated a peak corresponding to Glc_2_Man_9_GlcNAc_2_ (Figure 5) as well as minor amounts of Glc_1_Man_9_GlcNAc_2_ (data not shown), showing that *alg10-1* plants cannot perform the last step of the lipid-linked precursor biosynthesis.

### The *alg10-1* mutant displays a severe underglycosylation defect

**Figure 6:**
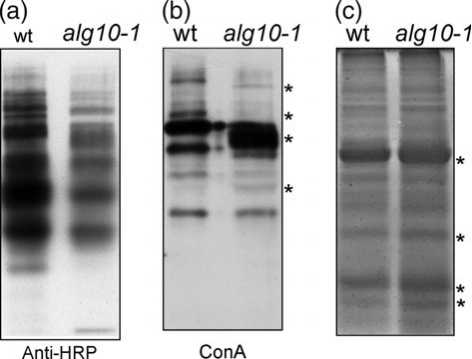
*alg10-1* displays differences in N-glycosylation. (a) Immunoblot analysis of total proteins extracted from wild-type (wt) and *alg10-1* leaves. Proteins were subjected to SDS-PAGE under reducing conditions and blots were analyzed using anti-horseradish peroxidase (anti-HRP) antibodies, which recognize complex *N*-glycans with a β1,2-xylose and core α1,3-fucose residues. (b) Proteins were subjected to SDS-PAGE under reducing conditions and blots were analyzed using the lectin concanavalin A (ConA). (c) Coomassie brilliant blue staining of total protein extracts. Asterisks indicate bands that differ between wild-type and *alg10-1*.

We asked whether the formation of incomplete lipid-linked oligosaccharides results in changes in N-glycosylation of proteins. Glycans were isolated from leaves and analyzed by matrix assisted laser desorption ionization time-of-flight mass spectrometry (MALDI-TOF-MS). The N-glycosylation profile of *alg10-1* was indistinguishable from the wild type, suggesting that the composition of *N*-glycans is not altered in the mutant (Figure S2). However, immunoblotting with antibodies against complex *N*-glycans revealed that the overall signal intensity was reduced in protein extracts from *alg10-1* compared with the wild-type (Figure 6a). Since the relative amounts of oligomannosidic and complex *N*-glycans were not altered in the mutant (Figure S2) this finding indicates underglycosylation with smaller amounts of complex *N*-glycans present on glycoproteins from *alg10-1*. A lectin blot with concanavalin A (ConA), which binds mainly to oligomannosidic glycans, also showed fainter signals in the mutant, and increased mobility of several glycoproteins was observed (Figure 6b). Differences in the mobility of bands were also found when protein extracts from seedlings were analyzed by SDS-PAGE and Coomassie staining (Figure 6c).

**Figure 7:**
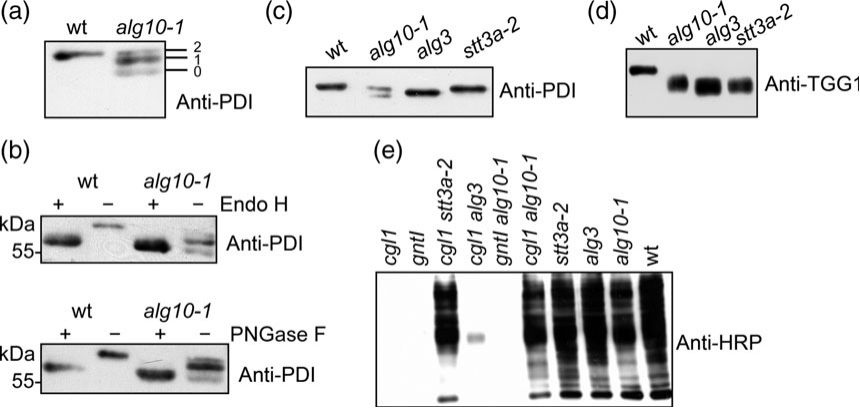
*alg10-1* displays a severe underglycosylation defect. (a) Immunoblot analysis of protein disulfide isomerase (PDI). Protein extracts were separated by SDS-PAGE and blots were analyzed with anti-PDI antibodies. The positions of the fully glycosylated PDI form (2), the form with one *N*-glycan (1) and the non-glycosylated PDI form (0) are given. (b) Protein extracts from wild-type (wt) and *alg10-1* were subjected to Endo H and PNGase F treatment to confirm the underglycosylation status of PDI in *alg10-1*. (c) Protein gel blot analysis of PDI from different underglycosylation mutants (*alg10-1*, *alg3*, *stt3a-2*). (d) Total proteins from wild-type, *alg10-1*, *alg3* and *stt3a-2* were separated by SDS-PAGE and blots were analyzed with anti-TGG1 antibodies. The shift in mobility of PDI and TGG1 in *alg3* is caused by the aberrant truncated *N*-glycan structures present in this mutant (Henquet *et al.*, 2008; Kajiura *et al.*, 2010). (e) Total proteins from the indicated single (*cgl1*, *gntI*, *stt3a-2*, *alg3*, *alg10-1*) and double mutants (*cgl1 stt3a-2*, *cgl1 alg3*, *gntI alg10-1* and *cgl1 alg10-1*) were analyzed with anti-horseradish peroxidase (anti-HRP) antibodies.

To analyze the underglycosylation defect of *alg10-1* in more detail we performed SDS-PAGE and immunoblotting using antibodies specific for different glycoproteins. Previous studies have analyzed the mobility of the ER-retained glycoprotein protein disulfide isomerase (PDI) to monitor underglycosylation defects in plants (Hoeberichts *et al.*, 2008; Kajiura *et al.*, 2010; Lerouxel *et al.*, 2005; Zhang *et al.*, 2009). In the *alg10-1* mutant three PDI forms were detectable while in the wild type a single PDI form was present (Figure 7a). Upon digestion with endoglycosidase H (Endo H) or peptide: *N*-glycosidase F (PNGase F) the three bands shifted to a band that migrated at the same position as the de-glycosylated wild-type protein, showing that PDI is underglycosylated in *alg10-1* (Figure 7b). Importantly, analysis of PDI forms present in different underglycosylation mutants revealed that ALG10 loss-of-function results in a more severe defect than observed for *alg3* and *stt3a-2* mutants as the underglycosylated PDI forms were more abundant in *alg10-1* (Henquet *et al.*, 2008; Kajiura *et al.*, 2010; Lerouxel *et al.*, 2005) (Figure 7c). ALG3 transfers the first mannose residue in the ER to the flipped dolichol precursor and the *alg3* null mutant displays only a very mild underglycosylation defect (Kajiura *et al.*, 2010). The shift in mobility of the major PDI form in *alg3* is not caused by underglycosylation but by the presence of truncated *N*-glycan structures in this mutant (Henquet *et al.*, 2008; Kajiura *et al.*, 2010). The *stt3a-2* mutant has a T-DNA insertion in the gene coding for the STT3A subunit of the oligosaccharyltransferase complex that results in a profound defect in glycosylation efficiency (Koiwa *et al.*, 2003).

Another glycoprotein that is sensitive to alterations in N-glycosylation is beta-thioglucoside glucohydrolase 1(TGG1) (Koiwa *et al.*, 2003; Zhang *et al.*, 2008, 2009). TGG1 is a vacuolar glycoprotein with nine potential N-glycosylation sites (Ueda *et al.*, 2006). On immunoblots probed with anti-TGG1 antibodies, TGG1 displayed a clear mobility shift corroborating our data that deficiency of ALG10 leads to hypoglycosylation of glycoproteins (Figure 7d).

Previously, it has been shown that the glycosylation defect in the *complex glycan 1* (*cgl1*) mutant can be rescued by crossing to the *stt3a-2* mutant (Frank *et al.*, 2008). The *cgl1* mutant contains a point mutation in the gene encoding *N*-acetylglucosaminyltransferase I (GnTI) that generates an additional N-glycosylation site and thus interferes with correct folding of GnTI and subsequent enzyme activity (Strasser *et al.*, 2005). As a consequence of reduced glycosylation, CGL1-GnTI becomes partially active in *stt3a-2* and the formation of complex *N*-glycans is restored (Frank *et al.*, 2008). To study if ALG10 deficiency has a similar effect on CGL1-GnTI we crossed *alg10-1* to *cgl1* and analyzed the formation of complex *N*-glycans by immunoblotting. In the *alg10-1 cgl1* double mutant complex *N*-glycan formation is restored, while in *alg10-1 gntI*, which contains a null allele of GnTI, no signal could be detected (Figure 7e). The staining intensity in the *alg10-1 cgl1* line was comparable to *stt3a-2 cgl1*, indicating similar degrees of CGL1-GnTI underglycosylation in the absence of ALG10 and STT3A, respectively. In contrast to *alg10-1*, glycosylation efficiency is only slightly affected in the *alg3* mutant (Figures 7e and S3).

Deficiency of N-glycosylation in the ER perturbs protein folding and quality control processes leading to ER stress and activation of the unfolded protein response (UPR) (Koizumi *et al.*, 1999; Koiwa *et al.*, 2003; Martínez and Chrispeels, 2003). We hypothesized that underglycosylation of proteins in *alg10-1* should lead to activation of the UPR. In accordance with our prediction, the expression of the folding chaperone binding protein (BiP) was increased in the *alg10-1* mutant (Figure S4) and a BiP2-promoter GUS construct (Oh *et al.*, 2003) expressed in *alg10-1* resulted in a strong GUS signal throughout the whole seedling (Figure S4) consistent with the finding that ALG10 loss-of-function leads to underglycosylation and subsequently to ER stress and activation of the unfolded protein response.

### The *alg10-1* mutant displays altered leaves and is more sensitive to salt stress

**Figure 8:**
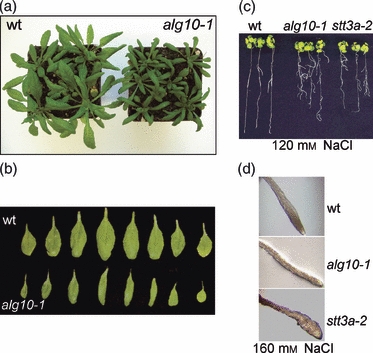
*alg10-1* mutant phenotypes. (a) Wild-type (wt) and *alg10-1* mutant plants grown on soil under long-day conditions (16-h light/8-h dark). (b) Leaf sizes of 6-week-old wild-type and *alg10-1* mutant plants. (c) *alg10-1* is salt sensitive. Seedlings were grown on MS medium for 6 days, transferred to new MS plates containing 120 mm NaCl and grown for 14 additional days. Col-0 wild-type and *stt3a-2* mutants were included for comparison. (d) Root tip of wild-type, *alg10-1* and *stt3a-2* seedlings grown for 14 days on MS medium supplemented with 160 mm NaCl.

Plants with ALG3 or STT3A deficiency display mild and severe underglycosylation defects, respectively, but do not display any obvious phenotype under normal growth conditions (Henquet *et al.*, 2008; Kajiura *et al.*, 2010; Koiwa *et al.*, 2003). The *alg10-1* seedlings were indistinguishable from the wild type when grown on MS medium, but *alg10-1* plants were smaller than the wild type and displayed alterations in leaf size when grown on soil (Figure 8a,b).

It was shown for some underglycosylation mutants that they are more sensitive towards salt stress and hypersensitive towards abscisic acid (ABA) and tunicamycin (Koiwa *et al.*, 2003; Zhang *et al.*, 2009). Root growth of *alg10-1* seedlings was affected when grown on media supplemented with 120 mm NaCl (Figure 8c). This effect was stronger when higher salt concentrations were used, while mannitol had only a very weak effect on the growth of *alg10-1* seedlings compared with the wild type (Figure S5). Interestingly, while *stt3a-2* displayed a swelling of the root tip when grown on MS medium with 160 mm NaCl, the root tip of *alg10-1* seedlings was indistinguishable from the wild type (Figure 8d). The *alg10-1* mutant was also more sensitive towards the treatment with tunicamycin than wild-type seedlings but less sensitive than *stt3a-2* (Figure S6). The *alg10-1* seeds germinated equally well on MS medium supplemented with ABA, but seedlings were slightly more ABA sensitive (Figure S7).

### Complementation of the Arabidopsis *alg10-1* mutant

Transgenic *A. thaliana* plants were generated by floral dipping of *alg10-1* plants with an *ALG10* construct, where *ALG10* expression was driven by the ubiquitin-10 promoter (*UBQ10:ALG10*), which provides consistent expression in *A. thaliana* tissues (Grefen *et al.*, 2010). Seedlings were selected on kanamycin and positive plants were screened by PCR for the presence of the transgene. Immunoblotting with anti-TGG1 and anti-horseradish peroxidase (anti-HRP) antibodies and analysis of lipid-linked oligosaccharides by LC-ESI-MS showed that ALG10 could fully complement the ALG10 deficiency of *alg10-1* (Figure S8). Moreover, the observed growth phenotype was also restored in the transgenic lines, confirming that these alterations are caused by the defect in lipid-linked oligosaccharide assembly and the resulting underglycosylation defect.

### The *alg10-1* mutant can rescue the embryo lethality of *knf-14*

**Figure 9:**
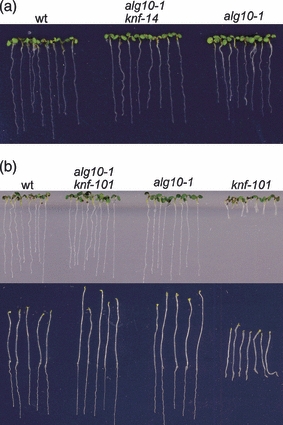
ALG10 deficiency suppresses the severe phenotypes of the *knf-14* and *knf-101* mutants which are deficient in α-glucosidase I activity. (a) The *alg10-1 knf-14* double mutant is indistinguishable from wild-type (wt) and *alg10-1* seedlings. Seedlings were grown on MS medium for 10 days (16-h light/8-h dark). (b) The *alg10-1 knf-101* double mutant has restored root elongation (10-day-old seedlings, 16-h light/8-h dark) and hypocotyl formation when grown in the dark for 7 days compared with *knf-101*.

Our data show that ALG10 adds the terminal glucose residue to the lipid-linked oligosaccharide. Based on these results we hypothesized that in the absence of the terminal glucose the activity of GCSI, which removes this residue, is not required for normal processing of *N*-glycans. As a consequence ALG10-deficient mutants might be able to rescue the defects observed for the *knf-14* GCSI loss-of-function mutant (Gillmor *et al.*, 2002) and for the weak *knf-101* allele (Furumizu and Komeda, 2008). To test our hypothesis we crossed *alg10-1* to *knf-14* as well as *knf-101* and screened by PCR and sequence analysis for putative double mutants. We could identify several lines, which were knockout for *alg10-1* and homozygous for the point mutations of *knf-14* or *knf-101*. Remarkably, the *alg10-1 knf-14* double mutant was viable and seedlings were indistinguishable from *alg10-1* and wild-type plants when grown on MS medium (Figure 9a). In addition, the root growth phenotype of light- and dark-grown *knf-101* seedlings was completely rescued in the *alg10-1 knf-101* mutant (Figure 9b). *N*-glycan analysis revealed that processing of *N*-glycans was completely restored in the *alg10-1 knf-14* and *alg10-1 knf-101* double mutants (Figures S9 and S10).

## Discussion

### Biosynthesis of the lipid-linked oligosaccharide precursor in plants

The first identified ALG glycosyltransferase from plants was *A. thaliana* ALG3 (Henquet *et al.*, 2008), which elongates the Man_5_GlcNAc_2_ precursor after flipping into the ER lumen by addition of one mannose residue. An ortholog of ALG11 catalyzing the biosynthetic step that precedes ALG3 on the cytosolic face and an ortholog of ALG12, which transfers the eighth mannose residue in the ER lumen, have also been characterized recently (Zhang *et al.*, 2009; Hong *et al.*, 2009). Here, we provide clear evidence that the identified *A. thaliana* ALG10 is an ortholog of the yeast ALG10 α1,2-glucosyltransferase: (i) *A. thaliana* ALG10 can complement the *S. cerevisiae Δalg10* mutant; (ii) the *alg10-1* loss-of-function mutant displays incomplete lipid-linked oligosaccharides resulting in an underglycosylation defect; and (iii) ALG10 deficiency suppresses the phenotypes of GCSI-deficient plants.

### The *alg10-1* underglycosylation defect

The assembly of the incomplete oligosaccharide precursor in *alg10-1* does not lead to detectable alterations of *N*-glycans. This finding is consistent with the fact that upon transfer by the oligosaccharyltransferase the glucose residues are immediately processed to generate monoglucosylated and oligomannosidic *N*-glycans (Hubbard and Robbins, 1979). Tri- and diglucosylated *N*-glycans are normally not detectable on glycoproteins from *A. thaliana* (Henquet *et al.*, 2008; Kajiura *et al.*, 2010; Strasser *et al.*, 2004) or other plant species (Wilson *et al.*, 2001). On the contrary the ALG10 deficiency results in a drastic underglycosylation of proteins in *A. thaliana*. A reduced *in vivo* glycosylation efficiency was also described for the yeast ALG10 mutant (Burda and Aebi, 1998) and can be explained by the reduced transfer of Glc_2_Man_9_GlcNAc_2_ to nascent polypeptides by the oligosaccharyltransferase (Karaoglu *et al.*, 2001; Murphy and Spiro, 1981; Turco and Robbins, 1979). To date the *S. cerevisiae* ALG10 is the only ALG10 protein that has been enzymatically characterized (Burda and Aebi, 1998). A putative rat ALG10 protein has been identified as a subunit of voltage-dependent K1 channels in rat brain, but a link to dolichol-linked oligosaccharide biosynthesis has not been established (Hoshi *et al.*, 1998). In humans, biosynthesis defects of the dolichol-linked oligosaccharide precursor are associated with diseases known as congenital disorders of glycosylation (CDG) (Haeuptle and Hennet, 2009). Interestingly, no ALG10-CDG patient has yet been identified and a mouse model for ALG10 deficiency has not been described (Thiel and Körner, 2011). Hence, the consequences of ALG10 deficiency for mammals are unknown. Here, we show that ALG10 loss-of-function in a multicellular organism has a profound effect on plant growth and tolerance to abiotic stress conditions. As the glycosylation process seems to be conserved in higher eukaryotes our data suggest that a similar ALG10 defect in mammals should also lead to drastic protein hypoglycosylation resulting in severe metabolic diseases or developmental changes in other organisms.

Here, we have established that several glycoproteins are underglycosylated in *alg10-1*. ALG10 deficiency has an effect on CGL1-GnTI, TGG1 and PDI as well as on some proteins detectable by ConA binding and Coomassie staining, indicating that a large number of glycoproteins are underglycosylated. Previously *A. thaliana* mutants that display reduced glycosylation efficiency and subsequent hypoglycosylation of proteins have been described (Hoeberichts *et al.*, 2008; Kajiura *et al.*, 2010; Koiwa *et al.*, 2003; Lerouxel *et al.*, 2005; Zhang *et al.*, 2008, 2009). These mutants have either a defect in the assembly of the lipid-linked core glycan or a deficiency in one of the oligosaccharyltransferase subunits. The *stt3a-2* mutant with a T-DNA insertion in the gene coding for the STT3a subunit of the oligosaccharyltransferase complex displayed underglycosylation of TGG1 (Koiwa *et al.*, 2003), PDI (Lerouxel *et al.*, 2005), CGL1-GNTI (Frank *et al.*, 2008) and the membrane-bound endo-1,4-β-glucanase KORRIGAN (Kang *et al.*, 2008; Zhang *et al.*, 2009). The *stt3a-2* mutant is viable, but hypersensitive to salt/osmotic stress (Koiwa *et al.*, 2003). Moreover, in *stt3a-2* (Koiwa *et al.*, 2003) and *alg10-1* mutants the UPR is activated and crossing of *alg10-1* to the weak KORRIGAN allele *rsw2-1* enhanced the *rsw2-1* root phenotype (data not shown) as was described for the *rsw2-1 stt3a-2* double mutant (Kang *et al.*, 2008), suggesting that KORRIGAN or another protein involved in cell wall synthesis is also subjected to hypoglycosylation in *alg10-1*. Despite these similarities there are, however, clear differences between *stt3a-2* and *alg10-1*: *stt3a-2* plants do not display any alteration of plant growth when grown on soil (Figure S11), but are more sensitive to salt stress and tunicamycin. It is very likely that these specific differences result from lack of N-glycosylation on a different group of glycoproteins.

Interestingly, a weak *A. thaliana* allele of the oligosaccharyltransferase subunit OST48/WBP1 (*dgl1-1*), which results in significant underglycosylation of PDI, but not of KORRIGAN, displays a severe growth phenotype at the seedling stage that finally leads to premature cessation of growth (Lerouxel *et al.*, 2005). The glycosylation capacity is also impaired in the *lew3* mutant, which is a weak *alg11* allele and shows a leaf-wilting phenotype as well as increased sensitivity to osmotic stress and ABA (Zhang *et al.*, 2009). The *lew3* plants are also hypersensitive to tunicamycin and show underglycosylation of PDI, but not of KORRIGAN. The comparatively mild underglycosylation defect present in the *alg3* null mutant (Kajiura *et al.*, 2010), which was confirmed by partial restoration of complex *N*-glycan formation in the *cgl1* mutant (Figure 7), does not result in any detectable growth phenotype or in increased salt stress sensitivity (Kajiura *et al.*, 2010) and the *alg12* null mutant does not display any protein hypoglyocsylation at all (Hong *et al.*, 2009). Together these data show that the different underglycosylation defects result in partially overlapping phenotypes (e.g. salt sensitivity in *stt3a-2*, *alg10-1* and *lew3*) but also in rather distinct phenotypes, which are presumably caused by the different degree of underglycosylation and by the different proteins that are affected in the mutants. In addition, some mutants like *lew3* generate aberrant truncated glycans, which are transferred to proteins and could influence the phenotype of the mutant. The characteristic leaf wilting phenotype of *lew1* and *lew3* mutants (Zhang *et al.*, 2008, 2009) was not found in the *alg10-1* plants. In summary our data suggest that the detected growth phenotype is specific for plants with ALG10 deficiency.

### Rescue of the lethality of the *gcsI* knockout mutant

The first step of *N*-glycan processing is the removal of the terminal α1,2-linked glucose from Glc_3_Man_9_GlcNAc_2_ in the ER by GCSI. One of the *knopf* mutants (*knf-14*) with a defect in cell expansion in early embryos is impaired in GCSI activity (Gillmor *et al.*, 2002). In another study a mutant with a T-DNA insertion in the *GCSI* gene displayed altered protein body formation, cell differentiation and embryo development (Boisson *et al.*, 2001). Moreover *knf-101*, which is a weaker allele, shows alterations of cell shape in epidermal cells (Furumizu and Komeda, 2008). All these studies propose a critical role for GCSI in plant development. However, our data show that these severe defects can be suppressed by ALG10 deficiency, suggesting that α-glucosidase I *per se* is not essential for embryo development and the lethality is caused by the presence of the terminal α1,2-linked glucose that blocks further trimming of the *N*-glycan. Mutants with defects in mannose trimming reactions are viable but display a severe root growth phenotype (Liebminger *et al.*, 2009), and impairment of maturation steps in the Golgi apparatus result in no or only conditional phenotypes (Kang *et al.*, 2008; Strasser *et al.*, 2004, 2007, 2006) suggesting that the critical step for the viability of plants is the removal of the second and third glucose residues by GCSII. *GCSII* T-DNA mutants have not been described in detail but the available data suggest that GCSII-null mutants are non-viable (Burn *et al.*, 2002; Soussilane *et al.*, 2009). Trimming of the first α1,3-glucose by GCSII is required to generate the monoglucosylated oligosaccharide that is specifically recognized by the lectins calnexin/calreticulin and thus enters the glycan-dependent protein folding and quality control cycle in the ER (D’Alessio *et al.*, 2010). In the absence of GCSI, subsequent trimming by GCSII is blocked and might lead to the observed embryo lethality. However, the null mutant of UDP-glucose:glycoprotein glucosyltransferase (Jin *et al.*, 2007), which performs the reglucosylation step that is required for prolonged interaction with the calnexin/calreticulin system, is viable, suggesting that a single round of calnexin/calreticulin binding is sufficient to provide efficient folding of the glycoproteins involved in cell wall synthesis and embryo development in *A. thaliana*. Apart from blocking glycoprotein folding, the presence of glucosylated oligosaccharides on certain glycoproteins could directly impair protein function, for example by preventing essential protein–protein interaction or enzyme activity. The importance of concerted deglucosylation is also highlighted by the fact that mammals have developed an additional glucosidase-independent pathway for removal of glucose residues which involves a Golgi-resident endo-α-mannosidase that releases Glc_1-3_Man from *N*-glycans and ensures that no proteins with glucosylated *N*-glycans are secreted (Zuber *et al.*, 2000). Moreover, malectin, an ER-resident protein that recognizes glucosylated oligosaccharides, has been found to participate in another backup quality control system in mammalian cells (Galli *et al.*, 2011; Schallus *et al.*, 2008). In summary these data show that glucose residues are critical determinants of protein glycosylation and quality control and their presence and controlled removal is crucial for the development of mammals and plants. Identification and characterization of other *N*-glycan biosynthesis mutants such as ALG5, which generates the Dol-P-glucose donor substrate for all ER-resident glucosyltransferases, ALG6 or ALG8 (Figure 1) are required to further dissect the role of the glucose residues on glycoproteins in plants. Moreover the *alg10-1* mutants are valuable tools to investigate the relationship between underglycosylation and plant growth as well as abiotic stress reactions.

## Experimental Procedures

### Plant material and growth conditions

*Arabidopsis thaliana* ecotype Columbia (Col-0), mutant plants and *N. benthamiana* were grown under long-day conditions (16-h light/8-h dark photoperiod) at 22°C as described previously (Liebminger *et al.*, 2009). The mutants *alg10-1* (SAIL_515_F10), *alg3* (SALK_064006), *gntI* (SALK_073560) (Kang *et al.*, 2008), *stt3a-2* (Koiwa *et al.*, 2003), *cgl1* (von Schaewen *et al.*, 1993) and *knf-14* (Gillmor *et al.*, 2002) were all obtained from the European Arabidopsis Stock Centre. The *knf-101* seeds were a kind gift of Yoshibumi Komeda (Department of Biological Sciences, The University of Tokyo, Tokyo, Japan) and BiP2:GUS seeds were kindly provided by Nozomu Koizumi (Nara Institute of Science and Technology, Nara, Japan).

The *alg10-1* T-DNA insertion was confirmed by sequencing of the PCR products obtained using primers ALG10_1F/RBsail1 and ALG10_2R/LBsail1 (see Table S1). Homozygous T-DNA insertion lines were identified using the primers ALG10_1F/_2R. The homozygous *alg3* T-DNA insertion line was identified by PCR using the primer combinations ALG3_9F/LBa1 and ALG3_9F/_8R. The other mutants were screened as previously described. Double mutants were generated by crossing and confirmed by PCR genotyping and subsequent sequencing. For the different treatments (e.g. NaCl) the seedlings were grown or incubated as described in the figures.

### RT-PCR analysis

Total RNA was purified from rosette leaves of *A. thaliana* wild-type plants and *alg10-1* using an SV total RNA isolation kit (Promega, http://www.promega.com/). First-strand cDNA was synthesized from 500 ng of total RNA at 42°C using oligo(dT) primers and AMV reverse transcriptase (Promega). The *ALG10* coding region was amplified with primers ALG10_4F/_5R using Turbo Pfu polymerase (Stratagene, http://www.stratagene.com/). The PCR product was subcloned using a ZERO Blunt TOPO PCR cloning kit (Invitrogen, http://www.invitrogen.com/) and sequenced using a BIG Dye Termination Cycle sequencing kit (Applied Biosystems, http://www.appliedbiosystems.com/). To detect *ALG10* transcripts, PCR was performed from cDNA using the primers ALG10_4F/_2R, ALG10_4F/_5R and ALG10_8F/_5R. Complementary DNA derived from the ubiquitin 5 (*UBQ5*) gene was amplified as a control using the primers UBQ5-D/-U. The PCR products were visualized by ethidium bromide staining.

### Subcellular localization of ALG10–GFP

The ALG10–GFP construct was generated by PCR amplification of the *ALG10* coding region using the primers ALG10_6F/_7R by Phusion High-Fidelity DNA Polymerase (Finnzymes, http://www.finnzymes.com/) and ligated into *Xba*I- and *Bam*HI-digested p20F plasmid (Schoberer *et al.*, 2009). Transient expression in *N. benthamiana* was done by infiltration of leaves as described previously (Schoberer *et al.*, 2009). For co-expression experiments, resuspended *Agrobacterium tumefaciens* strain UIA143 was diluted to an OD_600_ of 0.1 for ALG10–GFP and an OD_600_ of 0.05 for the ER marker protein GnTI-CaaaTS-mRFP (Schoberer *et al.*, 2009). Sampling and imaging of fluorescent proteins expressed in *N. benthamiana* leaves was performed 2 days after infiltration using a Leica TCS SP2 confocal microscope (http://www.leica.com/) as described in detail recently (Schoberer *et al.*, 2009).

### Yeast strains and expression in yeast cells

The *S. cerevisiae* wild-type strain BY4741 (*MATa his3D1 leu2D0 met15D0 ura3D0*) and the Δ*alg10* strain YGR227W (Y05880 *die2::kanMX4*) are from the EUROSCARF collection and were kindly provided by Gerhard Adam (BOKU-University of Natural Resources and Life Sciences, Vienna, Austria). The *kanMX4 alg10* disruption was confirmed by PCR using the following primer combinations: ScALG10_1F/Kan-B, Kan-D/Kan-C, ScALG10_4R/Kan-C and gene-specific primers ScALG10_1F/_2R and ScALG10_1F/_4R. Cells were grown in YPD broth or YPD agar (10 g L^−1^ yeast extract, 20 g L^−1^ peptone, 20 g L^−1^ dextrose, 20 g L^−1^ agar).

*ALG10* cDNA was amplified from wild-type *A. thaliana* (Col-0 ecotype) using the primers ALG10_10F/_11R. The *BgI*II- and *SaI*I-digested fragment was ligated into *Bam*HI- and *Xho*I-digested yeast vector pADHfw (kindly provided by Gerhard Adam), which provides expression under the control of the *ADH1* promoter. The resulting vector was transformed into the yeast Δ*alg10* strain using the lithium acetate procedure (Gietz *et al.*, 1992) and grown on plates prepared with yeast nitrogen base and yeast synthetic dropout media without leucine. Yeast transformants were analyzed by PCR using the primers ScALG10_1F/_4R, ScALG10_4R/Kan-C and ALG10_9R/pTk2-fw.

### Analysis of the carboxypeptidase Y glycosylation pattern

Yeast cells were grown in YPD broth medium at 30°C for 2 days and harvested by centrifugation at 16 000***g*** for 1 min. Cells were disrupted with glass beads using a Retsch mixer mill (http://www.retsch.com/) at 50–60 amplitude for 2 min and incubated in PBS for 15 min at 4°C. The soluble protein fraction was obtained by centrifugation at 8000***g*** and 10 000***g*** for 5 min, respectively. Protein concentration was measured with a BCA protein assay kit (Pierce, http://www.piercenet.com/) and proteins were separated by SDS-PAGE (8%) and blotted to a Hybond-ECL nitrocellulose membrane (GE Healthcare, http://www.gehealthcare.com/). After blocking with PBS containing 0.1% Tween 20 and 3% BSA for 60 min, blots were probed with monoclonal anti-CPY antibody (1:2.000 dilution in blocking buffer; Invitrogen) and developed using the Super Signal West Pico Chemiluminescent Substrate (Pierce).

### Preparation and analysis of lipid-linked glycans

Yeast cells (1 × 10^7^) were lysed in 2 ml of microsomal preparation buffer [50 mm 2-amino-2-(hydroxymethyl)-1,3-propanediol (TRIS)–HCl pH 7.3, 0.5 mm DTT, 1 mm EDTA, 250 mm sucrose and 0.5 mm phenylmethylsulfonyl fluoride (PMSF)] by shaking with glass beads for 2 h at 4°C. Cell lysates were separated from the glass beads, 3 ml of microsomal preparation buffer was added and the lysates were centrifuged at 8000***g*** for 20 min. The supernatant was centrifuged at 100 000***g*** for 75 min at 4°C. The resulting microsomal pellets assumed to contain most of the cells’ dolichol-linked precursor oligosaccharides were treated with 200 μl of 0.1 m trifluoroacetic acid (TFA) at 80°C for 1 h, which selectively hydrolyzes the labile sugar-phosphate linkage (M. Pabst *et al.,* unpublished).

In the case of seedlings, samples of approximately 1 g were minced in 5 ml of microsomal preparation buffer at 4°C with an Ultra-Turrax (IKA GmbH, Germany) disperser. Samples were centrifuged at 8000***g*** for 20 min at 4°C and the microsomal fraction was prepared and hydrolyzed as described above for yeast cells.

The hydrolyzed microsomal samples from yeast cells or plant seedlings were made slightly alkaline with ammonia and reduced by adding a twofold volume of a 2% sodium borohydride solution. Twenty-five per cent of the purified and vacuum-dried sample was injected to the liquid chromatography-MS analysis system. Liquid chromatography was done with a porous graphitized carbon column coupled to an electrospray ionization mass spectrometer (Q-TOF Global Ultima, Waters, http://www.waters.com/) for glycan detection as described recently (Pabst *et al.*, 2007, 2010). Peaks with the masses of doubly charged, reduced Glc_0-3_Man_9_GlcNAc_2_ were retrieved by simulated selected ion monitoring.

### *N*-glycan analysis by immunoblotting and lectin blots

Protein gel blot analysis of crude protein extracts was performed using anti-HRP antibody (1:10.000 diluted, Sigma-Aldrich, http://www.sigmaaldrich.com/) and peroxidase-conjugated concanavalin A (Sigma-Aldrich) as described (Schoberer *et al.*, 2009; Strasser *et al.*, 2004). Deglycosylation of proteins with peptide: *N*-Glycosidase F (PNGase F, New England Biolabs, http://www.neb.com/) and endoglycosidase H (Endo H, New England Biolabs) were done as described recently (Liebminger *et al.*, 2011).

### Protein gel blot analysis

Plant material was ground in liquid nitrogen, resuspended in 5–10 μl of PBS per mg of plant material, and centrifuged at 16 000***g*** for 10 min. An aliquot of the supernatant was immediately mixed with SDS-PAGE loading buffer, denatured at 95°C for 5 min, and subjected to SDS-PAGE (8 or 12%) under reducing conditions. Protein gel blots were blocked in PBS containing 0.1% Tween 20 and 3% BSA. The membranes were probed with anti-HRP, anti-PDI (1:5.000; custom-made antiserum raised against a peptide from *A. thaliana* PDI5 by Gramsch Laboratories, http://www.gramsch.de/) (Andème Ondzighi *et al.*, 2008), anti-BiP2 (1:1.500, Agrisera), anti-TGG1 (1:1.000, kindly provided by Ikuko Hara-Nishimura, Department of Botany, Graduate School of Science, Kyoto University, Kyoto, Japan).

### Total *N*-glycan analysis

Total *N*-glycan analysis was performed from 500 mg of rosette leaves or seedlings by MALDI-TOF-MS as described previously (Altmann *et al.*, 2001; Strasser *et al.*, 2004).

## SUPPORTING INFORMATION

TAIR annotation spreadsheet.

**Figure S1.** Multiple sequence alignment of *Arabidopsis thaliana* ALG10 with yeast (ScALG10) and human (HsALG10) proteins.

**Figure S2.** Matrix-assisted laser desorption ionization time-of-flight mass spectrometry (MALDI-TOF-MS) spectra of total *N*-glycans extracted from leaves of wild-type (wt) and *alg10-1* plants.

**Figure S3.** *alg10-1* displays a severe underglycosylation defect.

**Figure S4.** ALG10 deficiency activates the unfolded protein response.

**Figure S5.** Salt/osmotic stress sensitivity of *alg10-1*.

**Figure S6.** *alg10-1* seedlings are more sensitive to tunicamycin (TM) treatment than wild-type but not as hypersensitive as *stt3a-2*.

**Figure S7.** Phenotypic analysis of *alg10-1* under ABA treatment.

**Figure S8.** *ALG10* expressed under the *UBQ10* promoter can complement the phenotypic changes of the *alg10-1* mutant.

**Figure S9.** Matrix-assisted laser desorption ionization time-of-flight mass spectrometry (MALDI-TOF-MS) spectra of total *N*-glycans extracted from leaves of wild-type (wt) and *alg10-1 knf-14* double mutants.

**Figure S10.** Matrix-assisted laser desorption ionization time-of-flight mass spectrometry (MALDI-TOF-MS) spectra of total *N*-glycans extracted from leaves of *knf-101* mutants and *alg10-1 knf-101* double mutants.

**Figure S11.** Phenotypic comparison between wild-type, *alg10-1* and *stt3a-2* plants grown on soil.

**Table S1.** Oligonucleotide sequences used in this study.

**Data S1.** Methods – Histochemical analysis; complementation of the *alg10-1* mutant.

Please note: As a service to our authors and readers, this journal provides supporting information supplied by the authors. Such materials are peer-reviewed and may be re-organized for online delivery, but are not copy-edited or typeset. Technical support issues arising from supporting information (other than missing files) should be addressed to the authors.
