# Supplementary material for: Arabidopsis thaliana alpha1,2-glucosyltransferase (ALG10) is required for efficient N-glycosylation and leaf growth
Source: Plant J. 2011 Jul 27;68(2):314–25. doi: 10.1111/j.1365-313X.2011.04688.x (PMC3204403; doi:10.1111/j.1365-313X.2011.04688.x)
Supplement: Supplementary file 1 [file tpj0068-0314-SD1.pdf]

```
                                                   *********************
ALG10   -----------------------------------------MGKLAVAAITSLWVIPMSIIVNHIVPEPYMDEIFHVPQA  39
ScALG10 MDAKKNTGEANNDVLEEEAAIQLIAPGIARNLTQEVITGIFCNVVIYPLLLIYFVLTFRYMTTNIVPYEFIDEKFHVGQT  80
HsALG10 -----------------------------------MAQLEGYYFSAALSCTFLVSCLLFSAFSRALREPYMDEIFHLPQA  45

                            ********************* X               *********************
ALG10   QQYCNGNFRS--WDPMITTPPGLYYLSLAHVASLFPGMLLMENTSQSFSEACSTSVLRSTNAVSAVLCGVLVYEIIRFLG 117
ScALG10 LTYLKGKWTQ--WDPKITTPPGIYILGLINYYCIKP----------IFKSWSTLTILRLVNLLGGIIVFPILVLRPIFLF 148
HsALG10 QRYCEGHFSLSQWDPMITTLPGLYLVSIG---VIKPAIWIFG---WSEHVVCSIGMLRFVNLLFSVGNFYLLYLLFCKVQ 119

         X              ***************  ***************                 ***************
ALG10   PN---LSDRKATFMALVMSLYPLHWFFTFLYYTDVASLTAVLAMYLTCLKR------RYVLSALFGTLAVFIRQTNVVWM 188
ScALG10 N--------ALGFWPVSLMSFPLMTTYYYLFYTDVWSTILILQSLSCVLTLPFGPVKSIWLSAFFAGVSCLFRQTNIIWT 220
HsALG10 PRNKAASSIQRVLSTLTLAVFPTLYFFNFLYYTEAGSMFFTLFAYLMCLYG------NHKTSAFLGFCGFMFRQTNIIWA 193

        ******
ALG10   LFVACSGILDFTLDSSKQKGKQEVNQELHQSSNKKGATLRSNLRKRKSDISSDTSDPFNHGQTVPSTEDTSDLVYDIYTV 268
ScALG10 GFIMILAVERPAILQKQFN------------------------------------------------------THTFNNY 246
HsALG10 VFCAGNVIAQKLTEAWKTELQ------------------------KKEDRLPPIKGPFAEFR----------KILQFLLA 239

                      *********************           *********************
ALG10   ISTSWNLKWRILIKFSPFIFVVVAFGIFILWNGGIVLGAKEAHVVSLHFAQIMYFSLVSALFTAPLHFSVNQLRHQFHQL 348
ScALG10 LKLFIHAIDDFSNLVLPYMKNFVLFFIYLIWNRSITLGDKSSHSAGLHIVQIFYCFTFITVFSLPIWISRNFMKLYKLRI 326
HsALG10 YSMSFKNLSMLLLLTWPYILLGFLFCAFVVVNGGIVIGDRSSHEACLHFPQLFYFFSFTLFFSFPHLLSPSKIKTFLSLV 319

          X *********************                             ********************
ALG10   HRNWSLSLILTLVALVAGFVSVHFFSLAHPYLLADNRHYPFYLWRKIIN--AHWLMKYILVPVYVYSWFSILTLLAK--- 423
ScALG10 KRKPVQTFFEFIGIMLIIR----YFTKVHPFLLADNRHYTFYLFRRLIGNKSRLIKYFFMTPIYHFSTFAYLEVMRPNQL 402
HsALG10 WKRRILFFVVTLVSVFL----VWKFTYAHKYLLADNRHYTFYVWKRVFQ--RYETVKYLLVPAYIFAGWSIADSLK---- 389

                             *********************    ************                   ***
ALG10   ------------------TRRQTWILVYFLATCGVLVPTPLIEFRYYTIPFYLFMLHS-------------------CVR 466
ScALG10 TFHPITPLPIKEPVHLPIQLTHVSWTALITCTMVTIVPSPLFEPRYYILPYFFWRIFITCSCEPLIKDLKPAKEGENPIT 482
HsALG10 ------------------SKSIFWNLMFFICLFTVIVPQKLLEFRYFILPYVIYRLNI-------------------PLP 432

        ******
ALG10   SSSFATWLLIGTIFVSINVFTMAMFLFRPFKWSHEDGVQRFIW  509
ScALG10 ISSTKRLFMEFLWFMLFNVVTLVIFSKVSFPWTTEPYLQRIIW  525
HsALG10 PTSRLICELS--CYAVVNFITFFIFLNKTFQWPNSQDIQRFMW  473
```

Figure S1
