# Supplementary figures and images for: Arabidopsis thaliana alpha1,2-glucosyltransferase (ALG10) is required for efficient N-glycosylation and leaf growth
Source: Plant J. 2011 Jul 27;68(2):314–25. doi: 10.1111/j.1365-313X.2011.04688.x (PMC3204403; doi:10.1111/j.1365-313X.2011.04688.x)

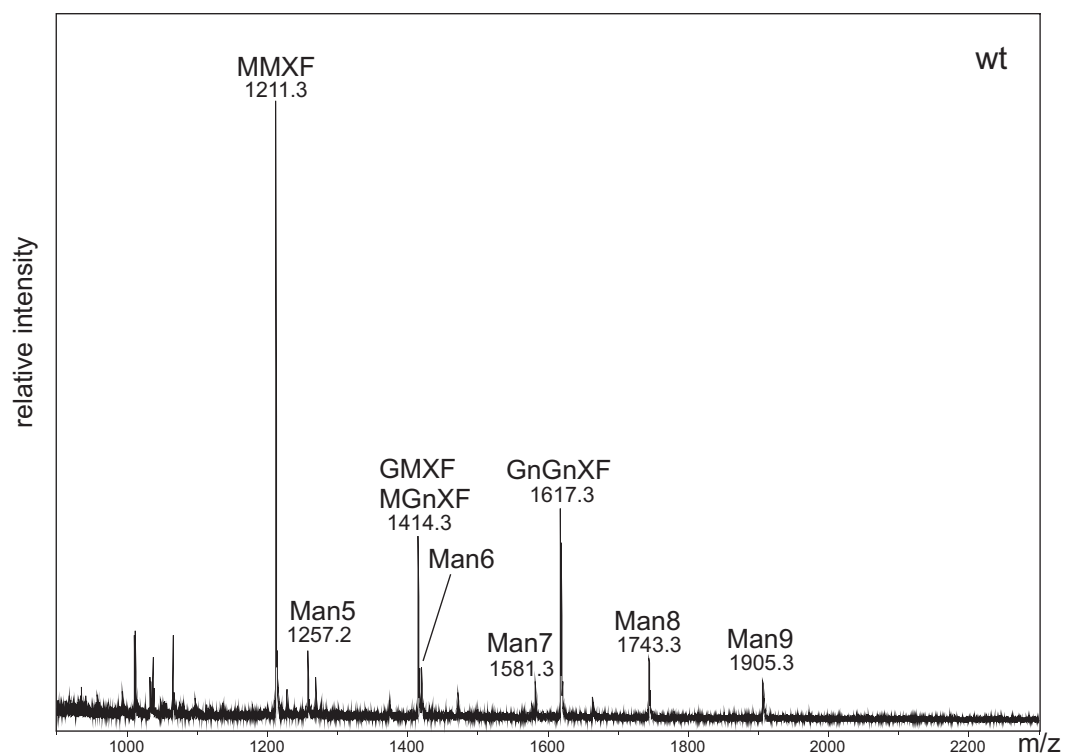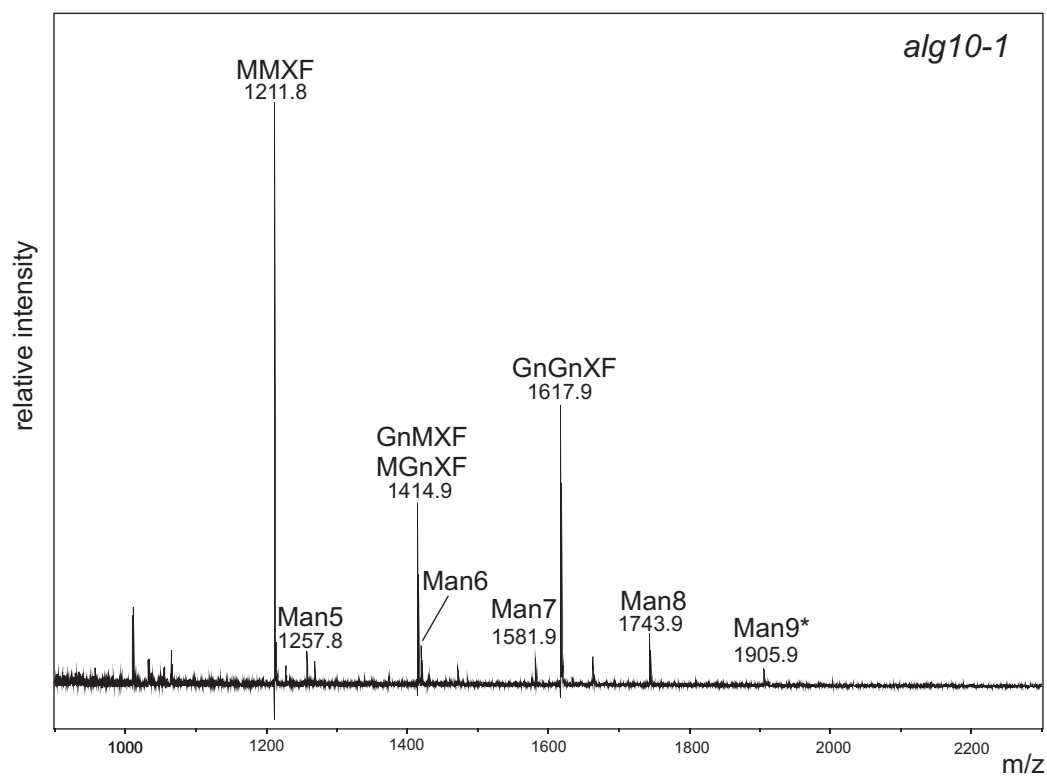

Figure S2

Supplement: Supplementary file 2 [file tpj0068-0314-SD2.pdf]

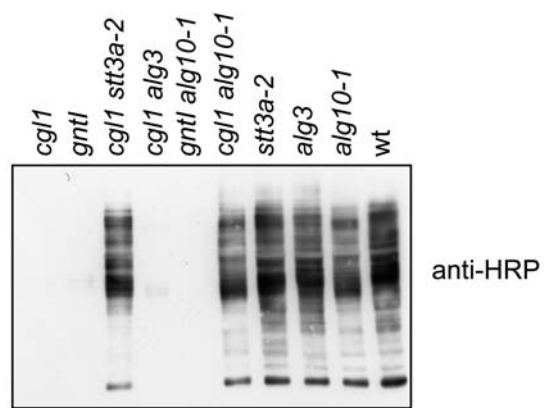

**Figure S3**

Supplement: Supplementary file 3 [file tpj0068-0314-SD3.pdf]

(a)

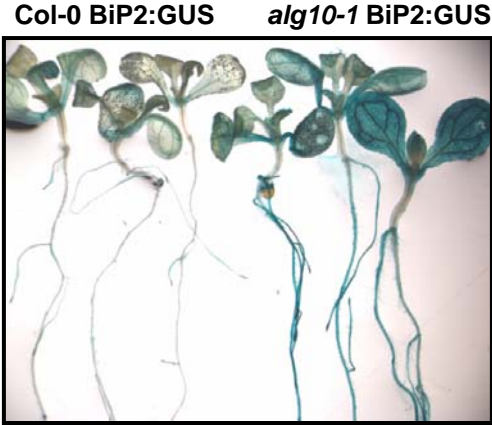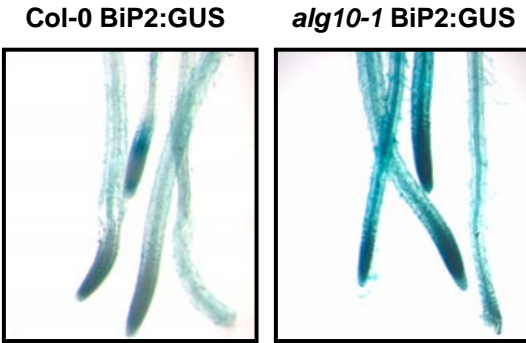

(b)

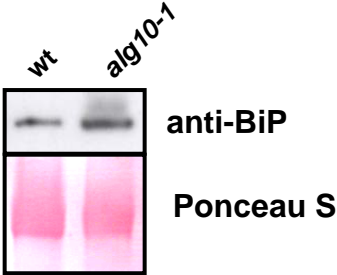

Figure S4

Supplement: Supplementary file 4 [file tpj0068-0314-SD4.pdf]

(a)

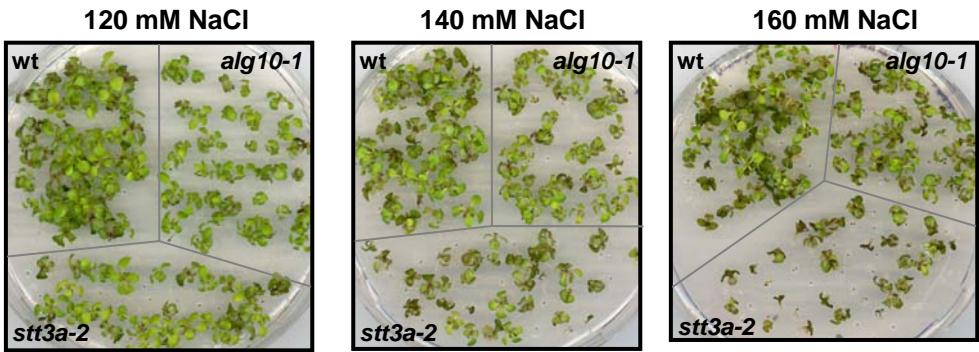

(b)

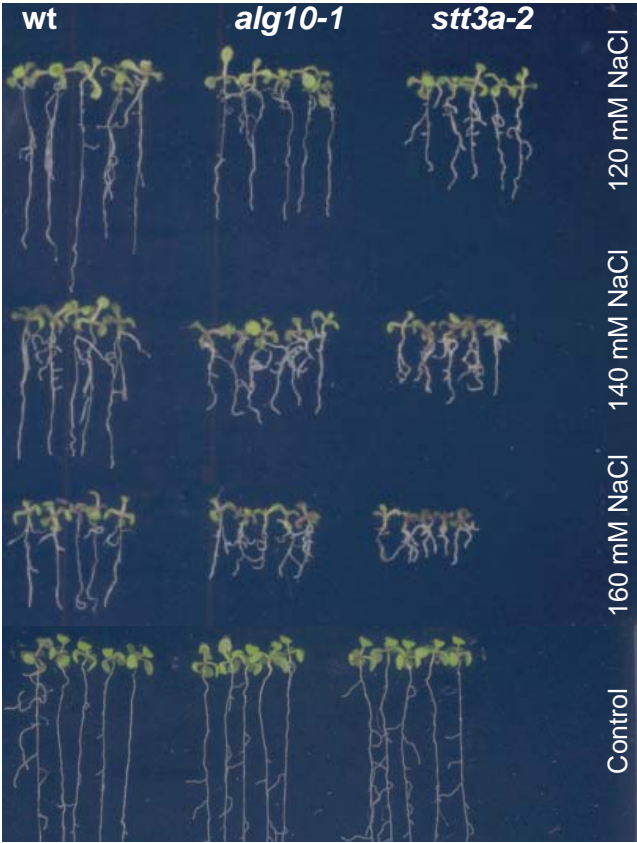

(c)

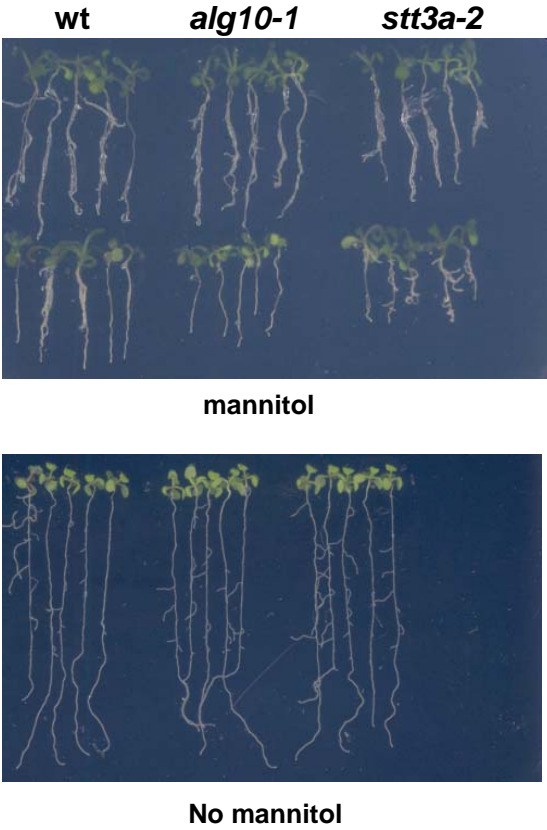

Figure S5

Supplement: Supplementary file 5 [file tpj0068-0314-SD5.pdf]

(a)

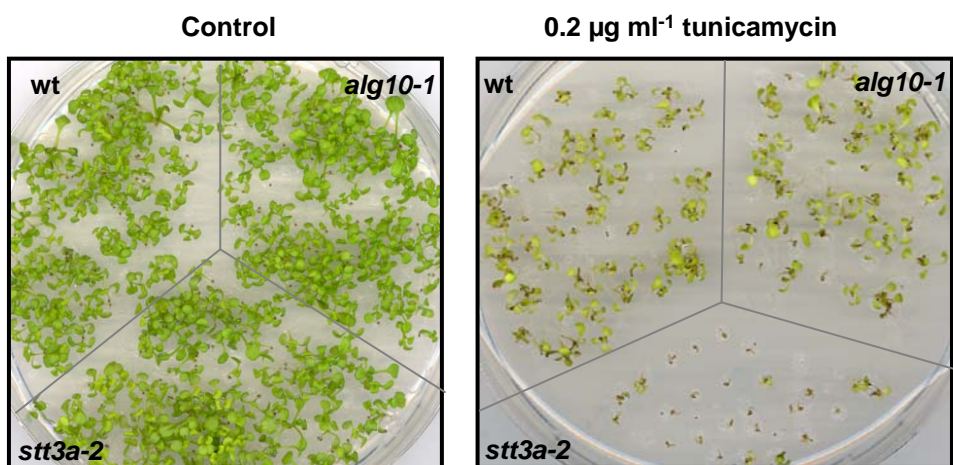

(b)

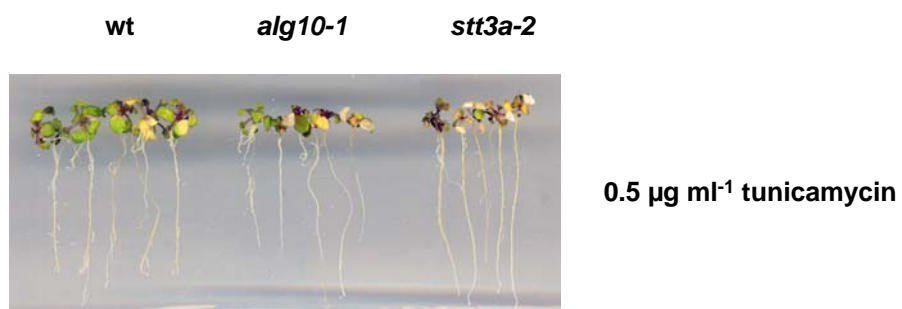

Figure S6

Supplement: Supplementary file 6 [file tpj0068-0314-SD6.pdf]

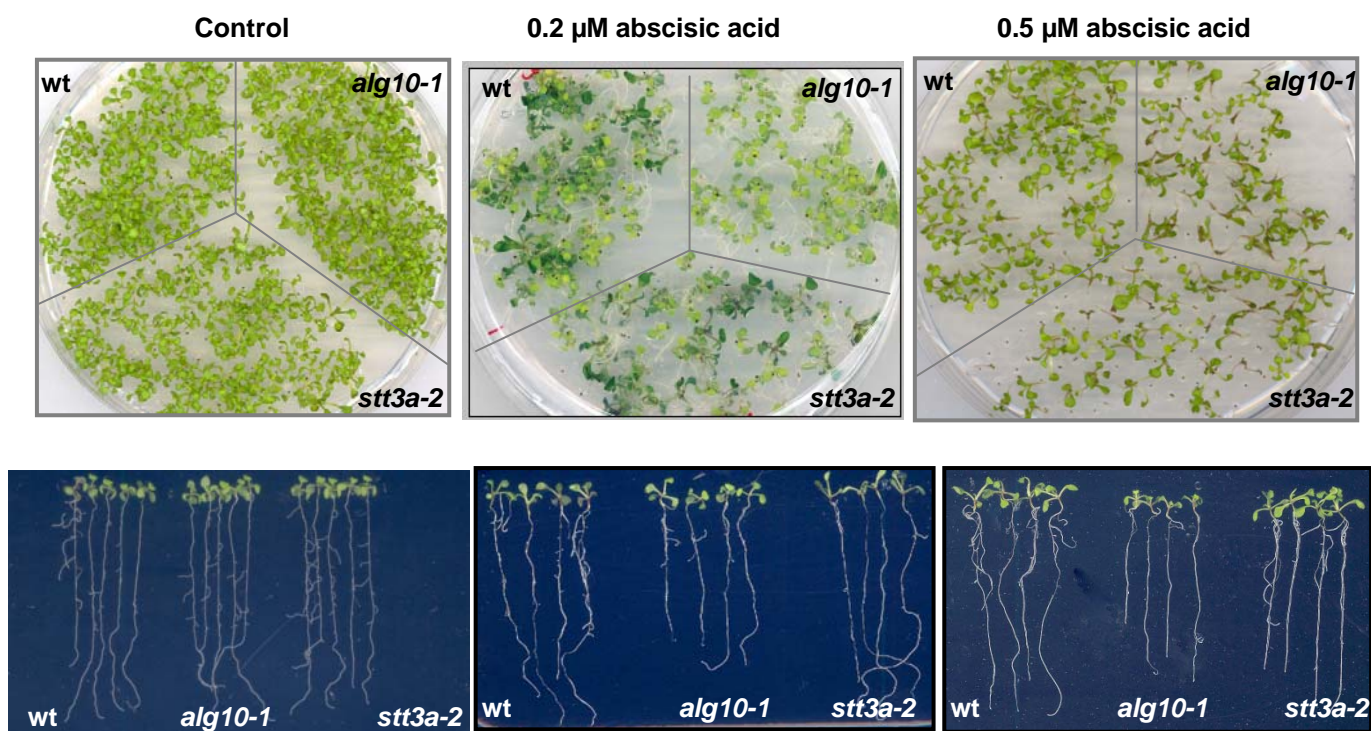

Figure S7

Supplement: Supplementary file 7 [file tpj0068-0314-SD7.pdf]

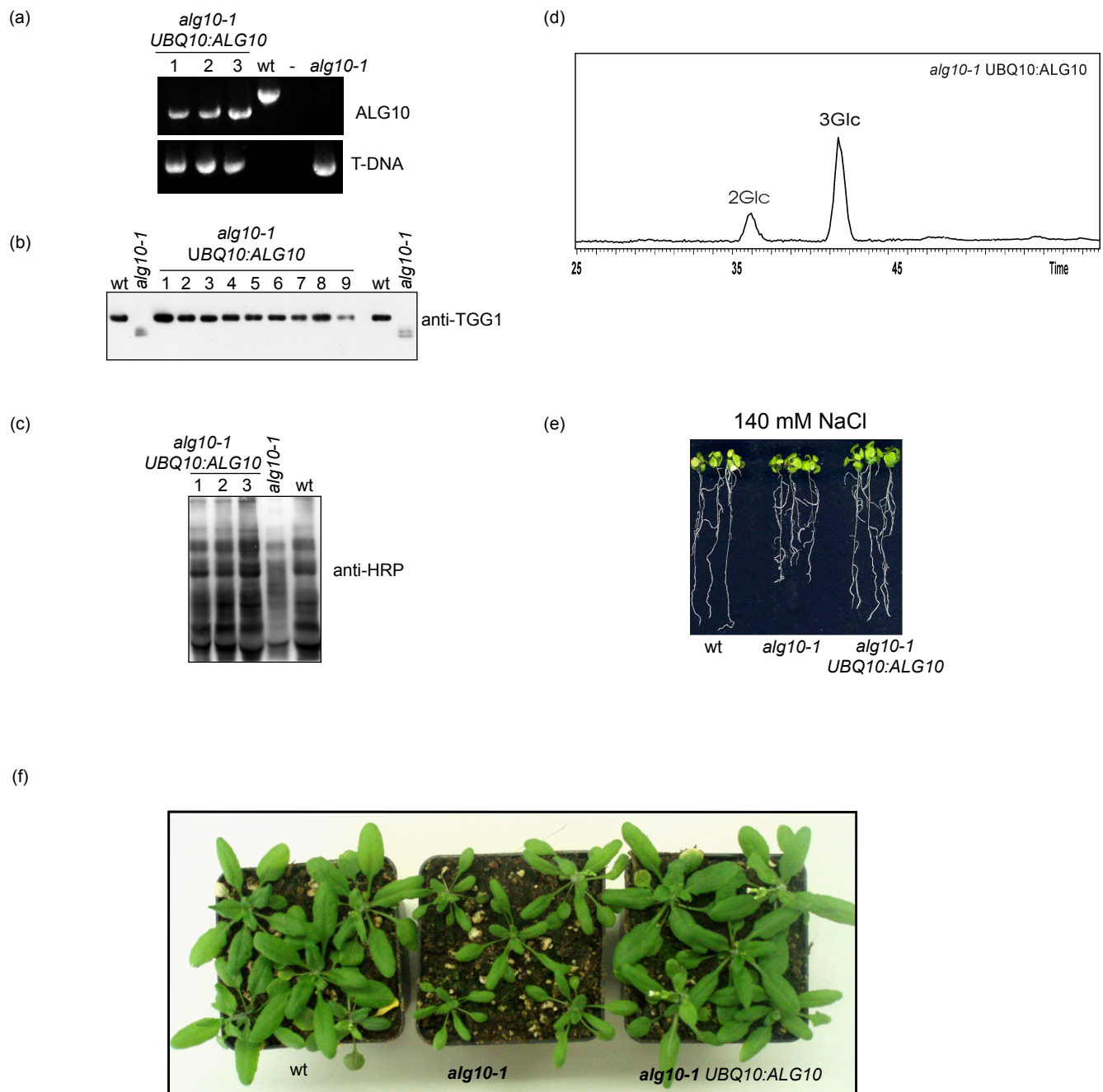

**Figure S8**

Supplement: Supplementary file 8 [file tpj0068-0314-SD8.pdf]

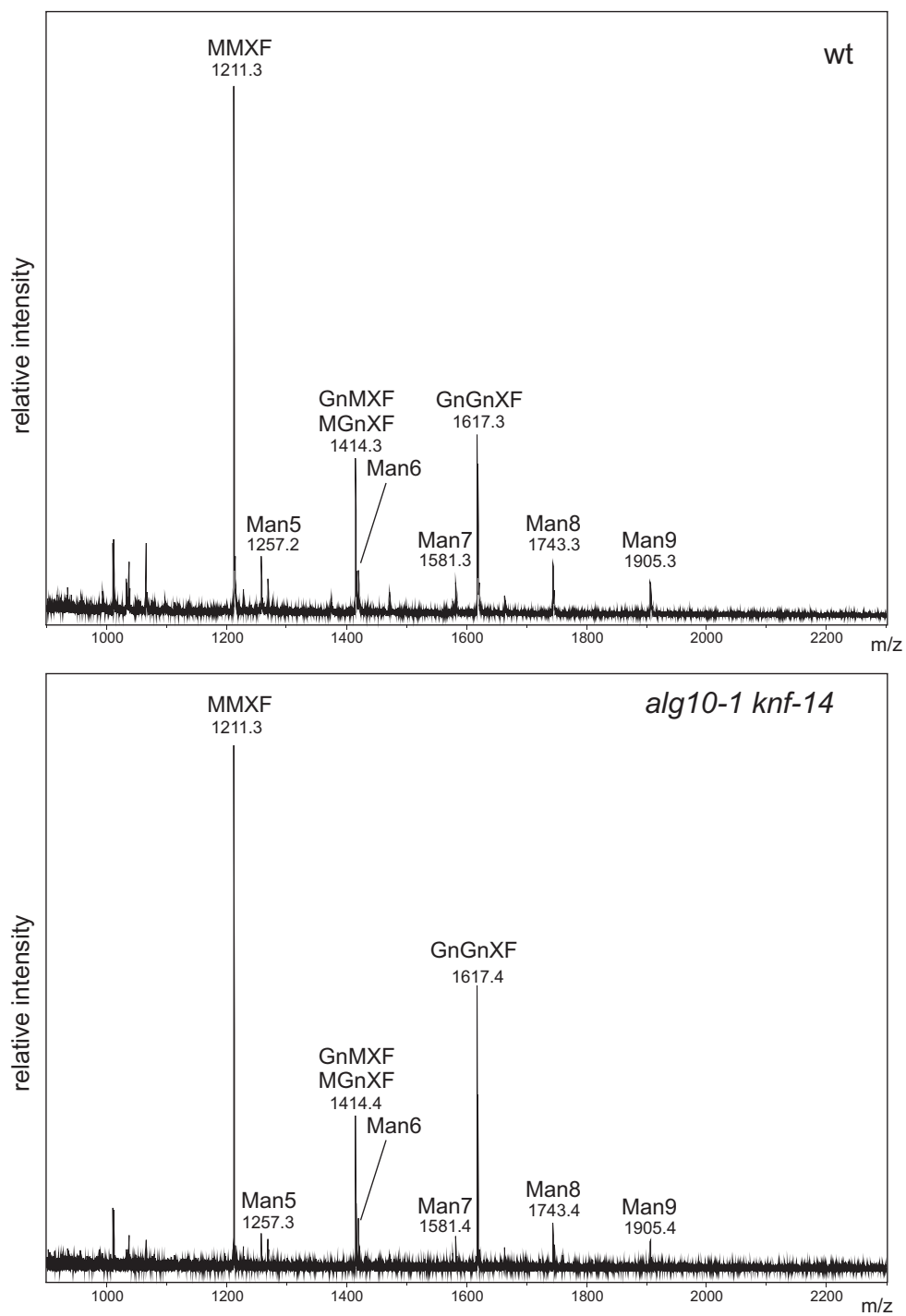

Figure S9

Supplement: Supplementary file 9 [file tpj0068-0314-SD9.pdf]

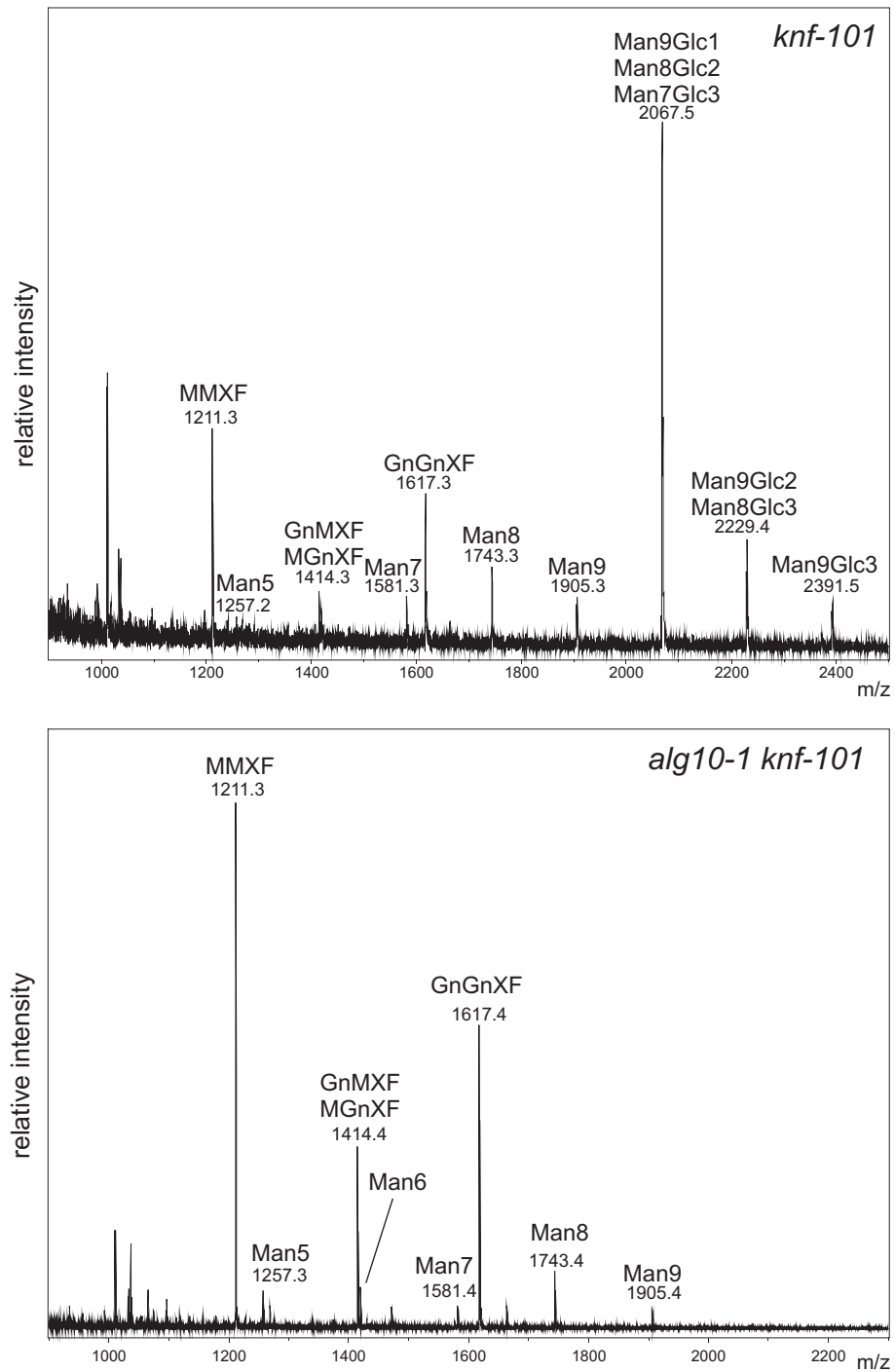

**Figure S10**

Supplement: Supplementary file 10 [file tpj0068-0314-SD10.pdf]

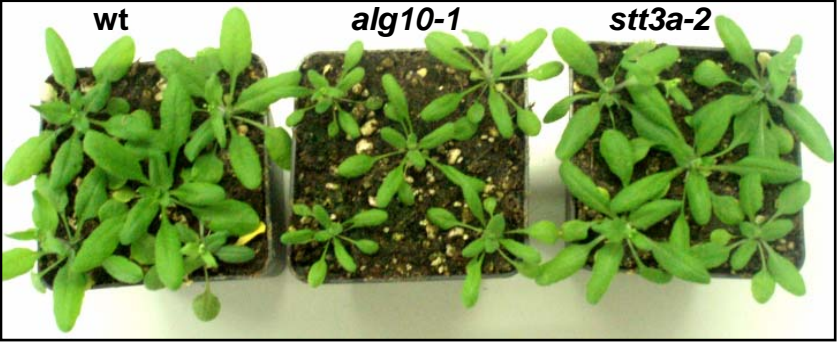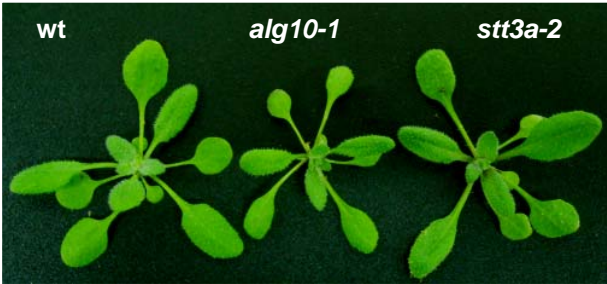

Figure S11

Supplement: Supplementary file 11 [file tpj0068-0314-SD11.pdf]
