# Supplementary material for: Arabidopsis thaliana alpha1,2-glucosyltransferase (ALG10) is required for efficient N-glycosylation and leaf growth
Source: Plant J. 2011 Jul 27;68(2):314–25. doi: 10.1111/j.1365-313X.2011.04688.x (PMC3204403; doi:10.1111/j.1365-313X.2011.04688.x)
Supplement: Supplementary file 12 [file tpj0068-0314-SD12.pdf]

Table S1

**Name**	**Sequences (5´- 3´)**
ALG10_1F	CAGTGCATTGGTAAGTGATTTGAAG
ALG10_2R	CTGAGAAGTGTAGGGGAGCAGTGA
ALG10_4F	GGATCCGATTGGTTCCTTTTGTG
ALG10_5R	GAGGAACCAAACAATAGCATCAAAAA
ALG10_6F	TGCTAGCATGGGGAAATTAGCCGTTGCAGCGA
ALG10_7R	TAGATCTCCATATAAACCTCTGGACACCATCTTC
ALG10_8F	GCTCTTTTCACTGCTCCCCTACAC
ALG10_9R	AGGGTAAAGCGACATGACTAACG
ALG10_10F	TAGATCTATGGGGAAATTAGCCGTTGCAGCGA
ALG10_11R	TGTCGACCTACCATATAAACCTCTGGACACCAT
ALG10_12F	CGTTAGTCATGTCGCTTTACCCTCTC
ALG10_24F	TATAACTAGTATGGGGAAATTAGCCGTTGCAGCGA
ALG10_25R	TATAAGATCTCTACCATATAAACCTCTGGACACCA
ALG3_9F	TGGTTTTGATTTGTCTGTTTATGATGG
ALG3_8R	CAAAGCAGACACCACTCCAATGAT
ScALG10_1F	TACACCGAGCCCAGTCACATC
ScALG10_2R	TGAAACGGGCCAAAAACCTAAG
ScALG10_4R	AAACGGTTGGCCACTAAAAGATG
Kan-B	CTGCAGCGAGGAGCCGTAAT
Kan-C	TGATTTTGATGACGAGCGTAAT
Kan-D	CCCCGGCAAAACAGCATTCC
RBsail1	CACACAGGAAACAGCTATGAC
LBsail1	TAGCATCTGAATTTCATAACCAATCTCGATACAC
LBa1	TGGTTCACGTAGTGGGCCATCG
pTK2-fw	TTCTGCACAATATTTCAAGCTATACC
UBQ5-D	AACCCTTGAGGTTGAATCATC
UBQ5-U	CTCCTTCTTTCTGGTAAACGT
