# Supplementary material for: Arabidopsis thaliana alpha1,2-glucosyltransferase (ALG10) is required for efficient N-glycosylation and leaf growth
Source: Plant J. 2011 Jul 27;68(2):314–25. doi: 10.1111/j.1365-313X.2011.04688.x (PMC3204403; doi:10.1111/j.1365-313X.2011.04688.x)
Supplement: Supplementary file 13 [file tpj0068-0314-SD13.doc]

**Figure S1.** Multiple sequence alignment of *A. thaliana* ALG10 with yeast (ScALG10) and human (HsALG10) proteins. The sequence alignments were performed with ClustalW (http://www.ch.embnet.org/software/ClustalW.html) and were edited manually. Conserved amino acid residues are shaded black and similar residues are shaded grey. Dashed lines represent gaps inserted for optimal alignment of the sequences.

**Figure S2.** MALDI-TOF-MS spectra of total N-glycans extracted from leaves of wild-type (wt) and alg10-1 plants. MS analysis was performed as described previously (Strasser et al., 2004; Liebminger et al., 2009).

**Figure S3.** *alg10-1* displays a severe underglycosylation defect. This figure represents a shorter exposure of the blot shown in Figure 7e.

**Figure S4.** ALG10 deficiency activates the UPR. (a) GUS activity is shown in whole seedlings and root tips. (b) Protein extracts from wild-type (Wt) and *alg10-1* plants were separated by SDS-PAGE and detected by immunoblotting with anti-BiP2 antibodies. Ponceau S staining is shown as a loading control.

**Figure S5.** Salt/osmotic stress sensitivity of *alg10-1.* (a and b) Wild-type, *alg10-1* and *stt3a-2* seeds were germinated on MS media supplemented with 120 mM, 140 mM and 160 mM NaCl and grown for 16 days under long day conditions. (c) Wild-type and *alg10-1* plants grown for 16 days in the presence of 300 mM mannitol (upper row) or 350 mM mannitol (lower row).

**Figure S6.** *alg10-1* seedlings are more sensitive to tunicamycin (TM) treatment than wild-type but not as hypersensitive as *stt3a-2.* (a) Seeds were germinated on MS medium supplemented with 0.2 µg ml^-1^ TM and grown for 3 weeks. (b) Seven-day-old seedlings were transferred to MS medium supplemented with 0.5 µg ml^-1^ TM and grown for 6 days. Leaves from *alg10-1* and *stt3a-2* seedlings were paler than leaves form wild-type seedlings.

**Figure S7.** Phenotypic analysis of *alg10-1* under ABA treatment. Seedlings were grown for 16 days on MS media supplemented with 0.2 µM and 0.5 µM abscisic acid (ABA). The *alg10-1* mutants were more sensitive to ABA than wild-type.

**Figure S8.** *ALG10* expressed under the *UBQ10* promoter (Grefen et al., 2010) can complement the phenotypic changes of the *alg10-1* mutant. (a) Presence of the *ALG10* cDNA construct in the transgenic lines was confirmed by PCR using gene-specific and T-DNA-specific primers. 3 independent transgenic lines are shown; wild-type and *alg10-1* were included as controls. Restoration of underglycosylation defects was confirmed by immunoblotting of protein extracts from transgenic *alg10-1* *UBQ10:ALG10* plants using (b) anti-TGG1 or (c) anti-HRP antibodies, which bind to complex N-glycans in plants. (d) MS analysis of lipid-linked oligosaccharides from *alg10-1* *UBQ10:ALG10* plants shows the restoration of Glc_3_Man_9_GlcNAc_2_ (3Glc) formation. 2Glc corresponds to the incompletely assembled Glc_2_Man_9_GlcNAc_2_ structure. (e) The transgenic *alg10-1* *UBQ10:ALG10* plants are indistinguishable from wild-type plants under salt stress conditions. One- week-old seedlings grown on MS media were transferred to MS media supplemented with 140 mM NaCl and grown for 2 weeks. (f) Phenotypic comparison of soil-grown wild-type, *alg10-1* and *alg10-1* *UBQ10:ALG10* plants.

**Figure S9.** MALDI-TOF-MS spectra of total N-glycans extracted from leaves of wild-type (wt) and *alg10-1 knf-14* double mutants. MS analysis was performed as described previously (Strasser et al., 2004; Liebminger et al., 2009).

**Figure S10.** MALDI-TOF-MS spectra of total N-glycans extracted from leaves of *knf-101* mutants and *alg10-1 knf-101* double mutants. MS analysis was performed as described previously (Strasser et al., 2004; Liebminger et al., 2009). The peak with a molecular mass of 2067.5 in the upper panel corresponds to a glycan with 10 hexoses and 2 N-acetylhexosamines. Three possible isobaric structures are indicated. Based on the described GCSI-defect in the *knf-101* mutant we propose that the most abundant glycan species corresponding to this mass should be Glc_3_Man_7_GlcNAc_2_ (Man7Glc3). The peak with a molecular mass of 2229.4 corresponds to a glycan with 11 hexoses and 2 N-acetylhexosamines. Two possible isobaric structures are given.

**Figure S11.** Phenotypic comparison between wild-type, *alg10-1* and *stt3a-2* plants grown on soil.

**Table S1.** Oligonucleotide sequences used in this study. Underlined regions represent restriction sites.

**Histochemical Analysis**

Five-day-old seedlings containing the BiP:promoter-GUS reporter were incubated overnight at 37°C in β-glucuronidase staining solution comprising 50 mM sodium phosphate pH 7.0, 10 mM EDTA, 0.1 % Triton X-100, 0.5 mg ml^-1^ X-Gluc and destained by washing with 70% ethanol similar as described (Jefferson et al., 1987)

**Complementation of the *alg10-1* Mutant**

For complementation of the *alg10-1* mutant, the ALG10 coding region was amplified from cDNA using primers ALG10_24F/_25R. The amplified fragment was *Spe*I and *Bgl*II digested and cloned into *Spe*I and *Bam*HI digested sites of binary expression vector pPT2M (Strasser et al., 2007). Subsequently the CaMV35S promoter of pPT2M was excised and replaced by the UBQ10 promoter (Grefen et al., 2010). Transgenic plants were generated by the floral dipping method and selected on MS medium supplemented with kanamycin. The presence of the ALG10 construct in the transgenic lines was confirmed by PCR using primers ALG10_12F/_2R, ALG10_8F/_11R and ALG_12F/RBsail1.

**References**

**Grefen, C., Donald, N., Hashimoto, K., Kudla, J., Schumacher, K., and Blatt, M.R.** (2010). A ubiquitin-10 promoter-based vector set for fluorescent protein tagging facilitates temporal stability and native protein distribution in transient and stable expression studies. *Plant J,* **64,** 355-365.

**Jefferson, R.A., Kavanagh, T.A., and Bevan, M.W.** (1987). GUS fusions: beta-glucuronidase as a sensitive and versatile gene fusion marker in higher plants. *EMBO J,* **6,** 3901-3907.

**Liebminger, E., Hüttner, S., Vavra, U., Fischl, R., Schoberer, J., Grass, J., Blaukopf, C., Seifert, G., Altmann, F., Mach, L., and Strasser, R.** (2009). Class I alpha-mannosidases are required for N-glycan processing and root development in *Arabidopsis thaliana*. *Plant Cell,* **21,** 3850-3867.

**Strasser, R., Bondili, J., Schoberer, J., Svoboda, B., Liebminger, E., Glössl, J., Altmann, F., Steinkellner, H., and Mach, L.** (2007). Enzymatic properties and subcellular localization of Arabidopsis beta-N-acetylhexosaminidases. *Plant Physiol*, **145,** 5-16.

**Strasser, R., Altmann, F., Mach, L., Glössl, J., and Steinkellner, H.** (2004). Generation of *Arabidopsis thaliana* plants with complex N-glycans lacking beta1,2-linked xylose and core alpha1,3-linked fucose. *FEBS Lett***, 561,** 132-136.
